# Global gene expression in granulosa cells of growing, plateau and atretic dominant follicles in cattle

**DOI:** 10.1186/s12958-015-0010-7

**Published:** 2015-03-08

**Authors:** Annie Girard, Isabelle Dufort, Gabriel Douville, Marc-André Sirard

**Affiliations:** Département des Sciences Animales, Pavillon INAF, Faculté des Sciences de l’Agriculture et de l’Alimentation, Centre de Recherche en Biologie de la Reproduction (CRBR), Université Laval, Québec, Québec, G1V 0A6 Canada

**Keywords:** Dominant follicle, Gene expression, Atresia, Granulosa cells

## Abstract

**Background:**

The physiological state of the dominant follicle is important as it may be linked to the competence of the oocyte within. The objective of this study was to analyze, by transcriptomic analysis, the changes occurring in granulosa cells from dominant follicles at different phases of follicular growth.

**Methods:**

Granulosa cells were collected from slaughterhouse dairy cattle follicles with a diameter greater than 9 mm, and were classified at different phases of follicle growth based on flow cytometry profiles of DNA content after staining with propidium iodide. Three phases were identified based on the proportion of cells in -G1 (less than 2n DNA), G0-G1 (2n DNA) or S-M (more than 2n DNA) and follicles were thus allocated to the growing, plateau or atresia group. Between group analysis (BGA) showed clear segregation of the three groups, and the groups were contrasted against each other in a loop design to identify differently expressed genes. Ingenuity Pathway Analysis (IPA) was used to identify the functions and upstream regulators associated with the observed differently expressed genes.

**Results:**

Major differences were observed between the growth phases. Granulosa cells from follicles in the plateau phase had increased expression of *TYRO3* and downregulation of *JAM2* compared to growing follicles, supporting the idea of a shift from proliferation to differentiation. On the other hand, genes regulating the response to oxidative stress (*VNN1*) and angiogenesis (*ANGPT2*) were upregulated in granulosa cells from atretic follicles. While the predicted activated functions in cells at the plateau stage compared to cells at the growing stage included synthesis and transport of molecules, the predictions for atretic follicles relative to plateau ones included an increase in apoptosis and cell death.

**Conclusion:**

Consistent with previous studies, these observations allowed us to match the presence of specific gene transcripts to a particular physiological status and consequently to classify follicles. The results also demonstrated that the plateau phase is not a simple ‘in between’ status between growth and atresia, as several characteristics are unique to this stage.

**Electronic supplementary material:**

The online version of this article (doi:10.1186/s12958-015-0010-7) contains supplementary material, which is available to authorized users.

## Background

Assisted reproductive technologies (ART) are an important part of reproduction management in humans and domestic animal species, as both have to cope with fertility problems. The dairy industry faces a decline in cows’ fertility, which brings additional costs to producers and lower profits [1]. Our understanding of the underlying causes of infertility is growing but many questions remain unanswered. As the ovarian follicle has a significant impact on oocyte quality [2] and the success of pregnancy, extensive research on follicular development is needed. The follicle is a dynamic structure and proceeds towards ovulation or atresia. Although we know some of the reasons why growth continues or stops, several key elements of the process remain unknown.

In the cow, the reserve of primordial follicles is estimated at 130,000 at birth [3]. However, most of them never reach ovulation: more than 99% will undergo atresia [4]. Ovarian follicles will take several months [5] to go from the primordial stage, which consist of an immature oocyte covered by a single layer of granulosa cells, to the antral stage, when the fluid-filled antrum forms. While activation of primordial follicles’ growth is a continuous process [6], recruitment of antral follicle cohorts follows a cyclical pattern. Cohorts are recruited in a wave-like manner in response to an increase in FSH levels [7] following either ovulation or regression of a dominant follicle. In cows, the number of follicles recruited in a wave varies between 2 and 50 [8] but can be increased by an artificial exaggeration of the FSH surge [9]. Selection of the dominant follicle occurs roughly 2.5 days after the FSH rise, at a diameter of approximately 8.5 millimetres [10]. FSH levels then drop and subordinate follicles slow their growth and undergo atresia, while the dominant follicle enters a phase of LH-dependent growth. It then reaches a plateau phase of non-exponential growth [11] with fewer cell divisions and a slower diameter increase. The presence of progesterone will determine the fate of this follicle: if this hormone is absent, the follicle will proceed to ovulation but if the progesterone level is high, the follicle will enter atresia [12]. Following the preovulatory LH surge and ovulation, the follicle undergoes drastic changes to become a highly vascular structure, the corpus luteum [13].

It is known that the developmental competence of the oocyte changes during the different phases of follicle development [14,15]. Although one would think that atresia is detrimental to the oocyte competence, a previous study has shown that bovine oocytes acquire competence late in folliculogenesis, during early atresia [16]. The same study suggested that early atresia could mimic some aspects of the pre-ovulatory environment, increasing the competence of the oocyte. The plateau phase result in oocytes with a high competence to reach the blastocyste stage [17], while the large growing healthy follicles have inferior developmental competence [18]. Given the impact of follicular status on oocyte competence, new tools to classify the follicles and identify the most physiologically mature ones are highly valuable.

Because different growing phases are associated with the expression of different genes, transcriptomic analysis of follicles can provide markers to determine their physiological status. Previous study on the transcriptomic profiles of granulosa cells revealed several genes linked to follicular development before establishment of dominance, and provided tools to classify medium-sized (6-9 mm) follicles in different growing phases [19].

The main objective of this study was to examine the transcriptomic profiles of dominant bovine follicles’ granulosa cells using microarrays to assess gene pathways associated with the three stages of follicular growth identified by flow cytometry (growing (G), plateau (P) and atretic (A) [20]), and secondary to suggest potential biomarkers for those stages.

## Methods

### Granulosa cell collection

Slaughterhouse ovaries from Holstein dairy cattle were kept on ice during transport and throughout the collection procedure in order to maintain RNA integrity. Reproductive status and estrous cycle stage of the animals are unknown. However, since the follicular waves still take place when a corpus luteum is present and progesterone levels are high, such as during the pregnancy or during the diestrus, this information is not essential for the purpose of the project. The growth and the plateau phases are present in dominant follicles whether progesterone is high or not; the only major difference is that in low progesterone environment, the follicle will become pre-ovulatory and may reach ovulation. Folliculogenesis is similar between pregnant and non-pregnant cows [21], with the only differences appearing around 3 days prior to ovulation [22] and being attributed to the selective growth and ovulation of the pre-ovulatory follicle in non-pregnant animals. In cattle, sizes reached by non-ovulatory dominant follicles are similar to those reached by ovulatory follicles one day before ovulation [21], and follicles from the first and second wave have an equivalent response to superovulation, including similar numbers of collected embryos [23]. Atretic dominant follicles most likely come from animals with high progesterone levels. Otherwise, the follicles would have reached the pre-ovulatory stage instead of progressing toward atresia.

Collection of the mural granulosa cell layer from individual follicles was performed by scraping and measures were taken to avoid contamination between samples, following the protocol of Douville et al. [14,19]. Briefly, the follicle diameter was measured with a ruler at the ovary surface. As follicular dominance in dairy cows is established at approximately 8.5 mm in diameter, only follicles >9 mm were selected. A portion of the follicular fluid was aspirated with a syringe and an 18-gauge needle in order to facilitate scraping. The hole made by the needle was widened by cutting out a triangular piece of ovarian epithelium using dissecting scissors. A small, round weighing spatula was then inserted through the hole and used to scrape the follicular walls gently to avoid collecting theca cells or blood contamination.

Granulosa cells from the 27 collected follicles were transferred to individual 1.5-ml centrifuge tubes containing 500 μl PBS solution without Ca^2+^ and Mg^2+^ (pH of 7.1 recipe (g/L): 0.2 KCL, 0.2 KH_2_PO_4_ , 8.0 NaCl, 1.14 Na_2_HPO_4_, 1.0 D-glucose (dextrose), 0.11 pyruvate (pyruvic acid)) passed through 0.22 μm filter and containing 125 mg/L EDTA (PBSE) [20]. The samples were vortexed gently (half speed for 5 seconds) and centrifuged at 600 × g for 30 seconds at 4°C. The supernatants were discarded to remove cellular debris or blood in the sample leaving approximately 10 μl in order to not disturb the pellets. Immediately after supernatant removal, the pellets were re-suspended by gentle vortexing in 50 μl of the same cold PBSE solution described above. 25 μl of each of the 50 μl suspensions were transferred to new 750 μl tubes on ice for propidium iodide (PI) staining and flow cytometry analysis. The remaining 25 μl was frozen for RNA extraction.

### Flow cytometry

The following procedures were done according to a previously published protocol [19]. Briefly, 250 μl of PBSE were added to the 25 μl suspension assigned to cytometry. Cold 95% EtOH was slowly added to the cell suspensions while vortexing at half-speed, to attain a final concentration of 70%. To permeabilize cell membranes, these suspensions were kept at 4°C for a minimum of 16 h. This allowed the DNA fragments resulting from apoptosis to exit the cells resulting in a lower DNA content detectable by flow cytometry [20,24]. All solutions were kept cold to avoid cell lysis. After centrifugation and removal of the alcohol containing DNA fragments, the samples were re-suspended in 500 μl of PI solution (50 μg/ml; Sigma-Aldrich Canada Ltd., Oakville, ON) in PBSE containing 0.1% Triton X-100 (Sigma-Aldrich Canada Ltd.), and RNase A (50 μg/ml, Sigma-Aldrich Canada Ltd.) [25]. The subsequent incubation period of 30 min at room temperature allowed the elimination of RNA by the RNase. The DNA was left as stained material within the cells.

Flow cytometry to analyze the DNA content of PI-stained cells was performed with a Beckman-Coulter EPICS XL (Mississauga, ON) equipped with a 488 nm laser. Forward light scatter (FSC) is an indicator of relative particle size and side light scatter is an indicator of granularity. Both were used to gate out debris during the acquisition process. Peak versus integrated fluorescence was used to gate out doublets which are two cells stuck together. Integrated fluorescence indicates the time a particle took to pass through the laser for a given event with two cells stuck together taking longer. Once the gates had been applied, 10,000 events were acquired.

The fluorescence histograms resulting from flow cytometry were modeled using the FlowJo software. The model provided the proportion of total events at the sub-G1 phase (G1-), corresponding to cells with less than 2n DNA content; at the G1 phase, corresponding to cells containing 2n of DNA; at the S phase, corresponding to cells that are duplicating their DNA material; and at the G2 phase, corresponding to cells having completely duplicated their genetic material to 4n.

The FlowJo software had its limitations as the model it applied used approximations and numbers were rounded. Consequently, the sum of the proportion of cells at each phase did not add up to exactly 100%, but varied between 92.83% and 110.54% for the samples collected in this experiment. The proportions were normalized by dividing the proportion of cells at a given phase by the sum of all proportions, and multiplying the result by 100.

To categorize the samples obtained from follicles >9 mm into groups representing physiological status, they were first ranked in decreasing order using the following formula:

$$ G2+S+G1-\left(G1-\right)=x $$

We collected 27 samples from 27 individual follicles greater than 9 mm in diameter. Two samples were excluded because of a low RNA integrity number (RIN). The remaining samples were separated in three groups based on the ***x*** value obtained with the formula above: seven samples with the highest ***x*** value were categorized as the “growing” (G) group; seven samples with the lowest ***x*** values were categorized as the “atretic” (A) group; and seven samples with intermediate ***x*** values (few mitosis and limited atresia) were categorized as the “plateau” (P) group. The four samples remaining which were at the boundaries between groups were not included in the rest of the analysis.

### RNA extraction and amplification

Total RNA extraction was performed using the *PicoPure RNA* Isolation kit, (Life Technologies Inc., Burlington, ON) under an RNase-free environment and including a DNase digestion (Qiagen, Toronto, ON) step. RNA quality and concentration were verified with a 2100 Bioanalyzer (Agilent, Santa Clara, CA), using RNA 6000 Nano reagents (Agilent). All hybridized samples had a RIN between 7.0 and 9.3. Using 5 ng of extracted total RNA as starting material, linear amplification of the mRNA fraction was performed using the RiboAmp HS^Plus^ RNA Amplification Kit (Life Technologies Inc., Burlington, ON) which relies on T7 RNA polymerase *in vitro* transcription (IVT) to yield antisense RNA (aRNA).

### Hybridization

Four aRNA samples (out of seven) from each condition were labelled with either Cy3 or Cy5 dyes using the ULS Fluorescent Labelling Kit for Agilent arrays (Kreatech Inc., Durham, NC). The labelled samples (825 ng) were prepared for hybridization using a Gene Expression Hybridization Kit (Agilent) step during which the Agilent spike was incorporated. The prepared samples were then hybridized onto Agilent-manufactured EmbryoGENE bovine microarray slides [26] in a loop design: growing against plateau (G vs P), plateau against atresia (P vs A) and growing against atresia (G vs A). The four selected granulosa samples originating from individual follicles in each category were hybridized individually against the four selected samples of the other categories, resulting in four biological replicates for each condition. For each contrast, a second slide was hybridized, inversing the color assigned to each condition, in order to produce a dye-swap, technical replicate. Hybridization was performed using Agilent hybridization chambers, in a rotating oven at 65°C for 17 h. This step was followed by a three minutes wash with GE Wash Buffer 1 (Agilent) at room temperature, a three minutes wash with GE Wash Buffer 2 (Agilent) at 42°C, a ten seconds wash with acetonitrile at room temperature and a 30 seconds wash with the Stabilization and Drying Solution at room temperature. The microarray slides were read by the Tecan’s PowerScanner with the Autogain procedure on each individual array. Images were then processed with Array-Pro Analyzer 6.4 (Media Cybernetics, Rockville, MD) to map each spot and to manually exclude spots obstructed by debris such as dust particles.

### Microarray statistical snalysis

Expression data was analyzed using the FlexArray software version 1.6.1 [27], which is based on the limma Bioconductor package [28]. Background subtraction was followed by loess within-array and quantile between-arrays normalization. The expression data was then fit to a linear model to estimate fold-changes and an empirical Bayes procedure was used to produce associated p-values.

### Analysis of differentially expressed genes

Genes to be investigated were selected based on a symmetrical raw fold change of 1.5 and a p-value < 0.05. The contrasts were set up in the following way: G vs P, P vs A and G vs A, where a positive fold change indicated upregulation in the second item of the contrast.

A between group analysis (BGA) [29] using R software [30] was performed in order to make a preliminary assessment of the efficiency of flow cytometry to separate the GC samples in relation to their follicular origin.

### Quantitative real-time PCR (qRT-PCR)

Gene expression profiles were validated using qRT-PCR. All the samples selected for each stage were used, resulting in seven biological replicates per category. Therefore, for each category, qRT-PCR validation was performed on the four samples selected for hybridization and on the other three samples best characterizing the given growth phase, as determined by flow cytometry.

Quantitative RT-PCR was performed on non-amplified material from all seven samples per group. Total extracted RNA was reverse transcribed with oligo-dT primers and the qScript Flex cDNA Synthesis Kit (Quanta Biosciences, Gaithersburg, MD). Primers specific to the targeted transcript were designed using PrimerQuest (Integrated DNA technologies, Coralville, IA). The qRT-PCR analysis was performed using the LightCycler 480 SYBR Green I Master and the LightCycler 480 System (Roche, Laval, Qc, Cdn). PCR products were purified with the QIAquick®PCR Purification Kit, and diluted from 1 × 10^−4^ ng to 1 × 10^−8^ ng to calculate a five-point standard curve in order to quantify the reaction output. Data normalization was performed based on geNORM normalization [31] using two reference genes that are well suited for dominant follicles: eukaryotic translation initiation factor 2B, subunit 2 beta (*EIF2B2*) and splicing factor 3a, subunit 1 (*SF3A1*) [32]. Purified PCR products were sequenced to confirm specificity. Primer sequences, product sizes, annealing temperatures, and accession numbers are available in Additional file 1: Table S1.

### qRT-PCR statistical analysis

GraphPad Prism 5.02 (GraphPad Software Inc., San Diego, CA) was used to perform statistical analysis on qRT-PCR data. One-tailed t tests were performed to assess the differences between the samples of each contrast. A parametric one-way analysis of variance (ANOVA) was performed with a Newman-Keuls post–hoc test at a significance level alpha = 0.05. A post-test for linear trend and a Bartlett’s test for equal variance were also performed for each set of data. When variances differed significantly (p-value < 0.05) within a set, the data were transformed using the equation Y = log (y * 1e^12^) to obtain more homogenous variance. A large multiplication factor was used to obtain values greater than one, as the logarithm of numbers between 0 and 1 would give a negative result.

### Ingenuity Pathway Analysis

The Ingenuity Pathway Analysis (IPA) software was used to further identify functional attributes of the differentially expressed transcripts and to uncover the interactions between the differentially expressed genes within the dataset and with other molecules in the IPA database. The limma data for all 38,732 probes on the EmbryoGENE bovine array targeting a transcript for each contrast (G vs P, P vs A and G vs A) were uploaded as individual dataset into the IPA software. A “core analysis” was performed separately for each contrast using default parameters. The cut-off values were set at 1.5 and 0.05 for the fold change and p-value respectively.

The *Functions* results of the IPA core analysis not only allowed transcripts to be grouped under particular functional annotations, but were also used to determine which functions were likely increased or decreased by integrating the direction of the fold change of a particular molecule and its documented impact on that function in the literature. IPA calculates a z-score for each functional category where a positive z-score predicts that the biological process is trending towards an increase, and a negative z-score corresponds to a decreasing trend. Z-scores ≥ 2 or ≤ −2 indicates that the function’s trend is statistically significant. The p-value, calculated by Fisher’s Exact Test, measures the likelihood that the genes in a dataset could be randomly associated to a given function.

The *Upstream analysis* section of the core analysis was used to determine which upstream regulators were likely activated or inhibited in each contrast. The *My Pathway* tool was used to construct gene interaction networks in a schematic form.

## Results

### Follicular size

The mean follicular size was not different between the three groups (ANOVA p–value = 0.845). The means and standard deviations for the growing, plateau and atretic groups were 14.9 ± 3.8 mm, 15.4 ± 4.8 and 14.1 ± 3.8 mm, respectively.

### Microarray

**Figure 1:**
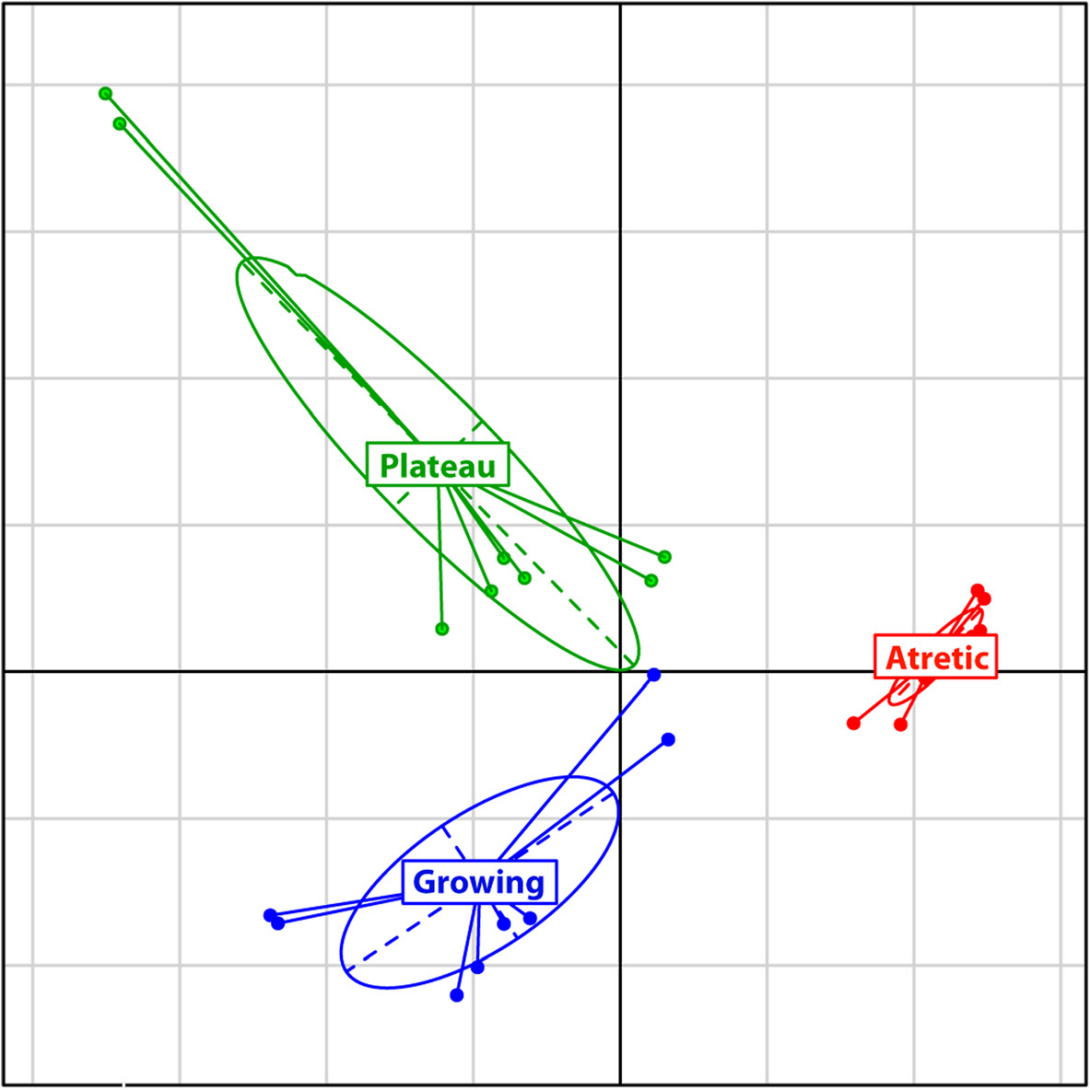
**Between group analysis (BGA).** The analysis was performed with all probes’ data in the green colour, using R software to show the three growth stages: Growing (blue), Plateau (green) and Atretic (red). Given the loop design of the experiment, 2 sets of data were available for each array, for a total of 8 data sets per growth stage.

The data discussed in this publication have been deposited in NCBI’s Gene Expression Omnibus [33] and are accessible through GEO Series accession numbers GSE63904, GSE63918 and GSE63919. Between group analysis demonstrated that the samples were segregated into three distinct groups (Figure 1). The position of the plateau group between the two other groups on the horizontal axis but above them on the vertical axis indicated that this phase has its own characteristics and is more than a simple intermediate stage between two physiological states.

**Figure 2:**
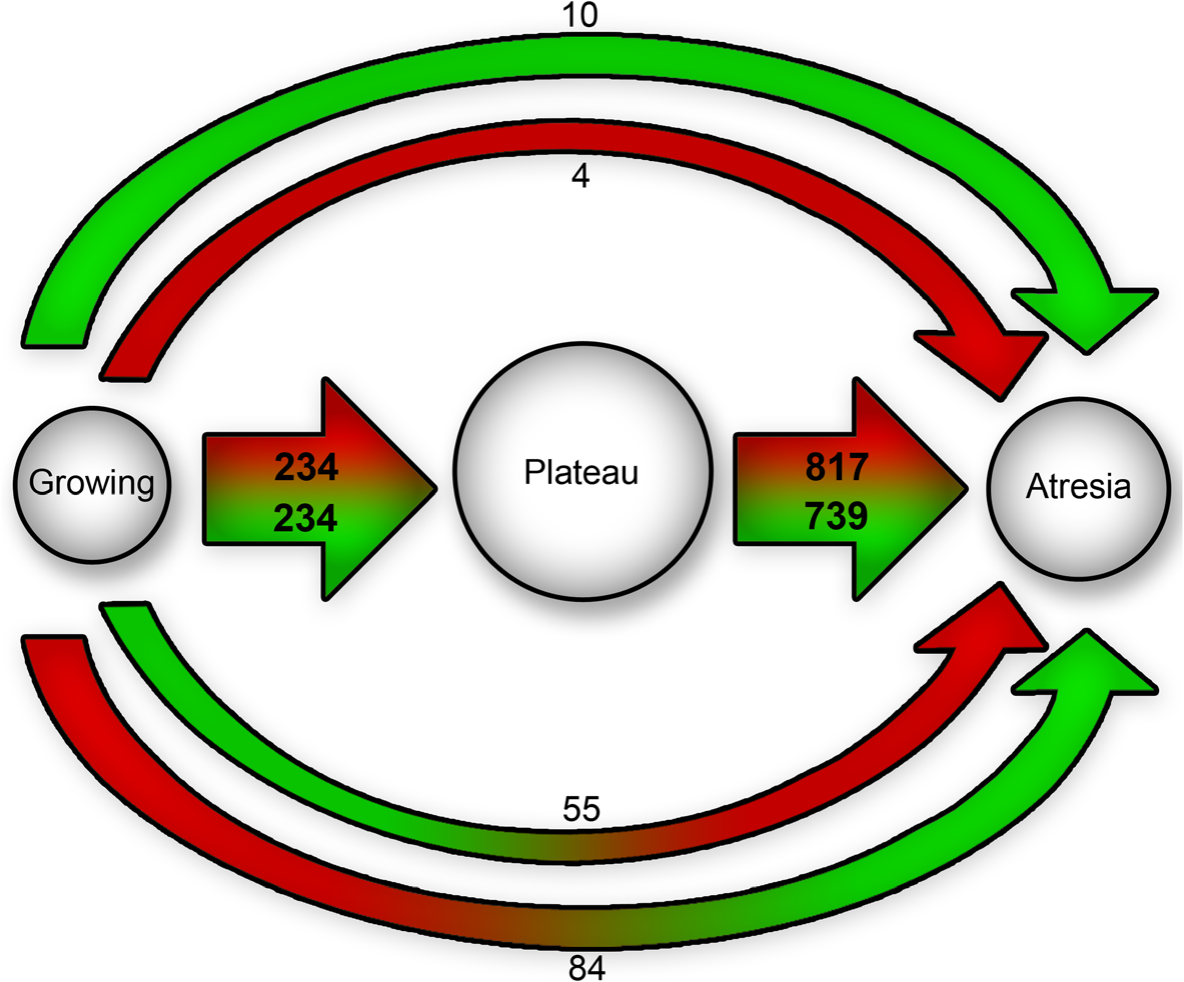
**Number of differently expressed genes (DEGs).** Diagram of the number of genes modulated between the growth stages, where a different expression was defined as a symmetrical fold change of more than 1.5, a minimal intensity of 7 and a p-value < 0.05. Red represents upregulated genes while green represents downregulated ones. The arrows at the top of the figure illustrate the genes that are regulated in the same way during all the transitions; the ones in the middle represent the genes that are upregulated (red part of the arrow) or down regulated (green part) only during one specific transition; the arrows at the bottom represent the genes that undergo a dramatic shift in their regulation (from downregulation to upregulation, or vice versa). The numbers accompanying the arrows show the number of genes in each category.

A cut-off at a symmetrical fold change of 1.5 and at a p-value of 0.05 was used after normalisation and resulted in 761 differentially expressed genes (DEG) between the growing and the plateau phase, of which 468 were specific to that transition. Between the plateau and the atretic phase, 1,709 genes were differentially expressed with 1,556 of them being specific to that transition. There were 153 genes modified in both contrasts, but only 14 of them underwent continuous change between the three states (Figure 2).

### qRT-PCR

The 25 genes selected for quantification by qRT-PCR were: *ACE2, ANGPT2, ANK3, ANKRD1, APOA1, BMP4, BUB1, CCNB1, CD36, CKS2, JAM2, MT2A, NMB, NR4A1, NRP1, PRC1, PTTG1, RARRES1, RELN, SERPINE1, STAR, TRIB2, TUBB6, TYRO3* and *VNN1* (see Additional file 2: Table S2 for full names). The gene *CYP17A1*, which is not expressed in granulosa cells, was also quantified to assess the potential contamination with theca cells. The expression level of this gene was similar in most samples, except for one of the atretic phase samples (A2) where it was higher, suggesting a considerable contamination with theca cells. The variance between the atretic samples was initially significantly different (p-value < 0.0001) for the expression level of *CYP17A1* but with the exclusion of sample A2, the Bartlett’s test for equal variance was not significant (p-value = 0.5323), showing an improved variance homogeneity. Thus, sample A2 was removed from the analysis.

The expression level of aromatase (*CYP19A1*) was also quantified to assess the steroidogenic activity of the granulosa cells. The mean expression level and variance was not different at any of the physiological stages (p-value > 0.1). The homogenous expression level of aromatase suggested that all the samples originated from non pre-ovulatory follicles [34]. This homogenous expression of CYP19A1 goes against previous studies, that instead observed a decrease in atretic follicles [35]. On the other hand, a sharp decrease in aromatase was also observed in subordinate follicles when compared to dominant ones [36], and aromatase activity in dominant atretic follicles do not decrease as fast as the concentration of estradiol in follicular fluid [37,38]. Taken together, these results suggest that dominant follicles may not behave in the same way as subordinate ones, and that aromatase activity may not change rapidly enough to give significant differences that would better reflect the changes observed in estradiol concentrations. More studies would be needed to clarify that point.

**Figure 3:**
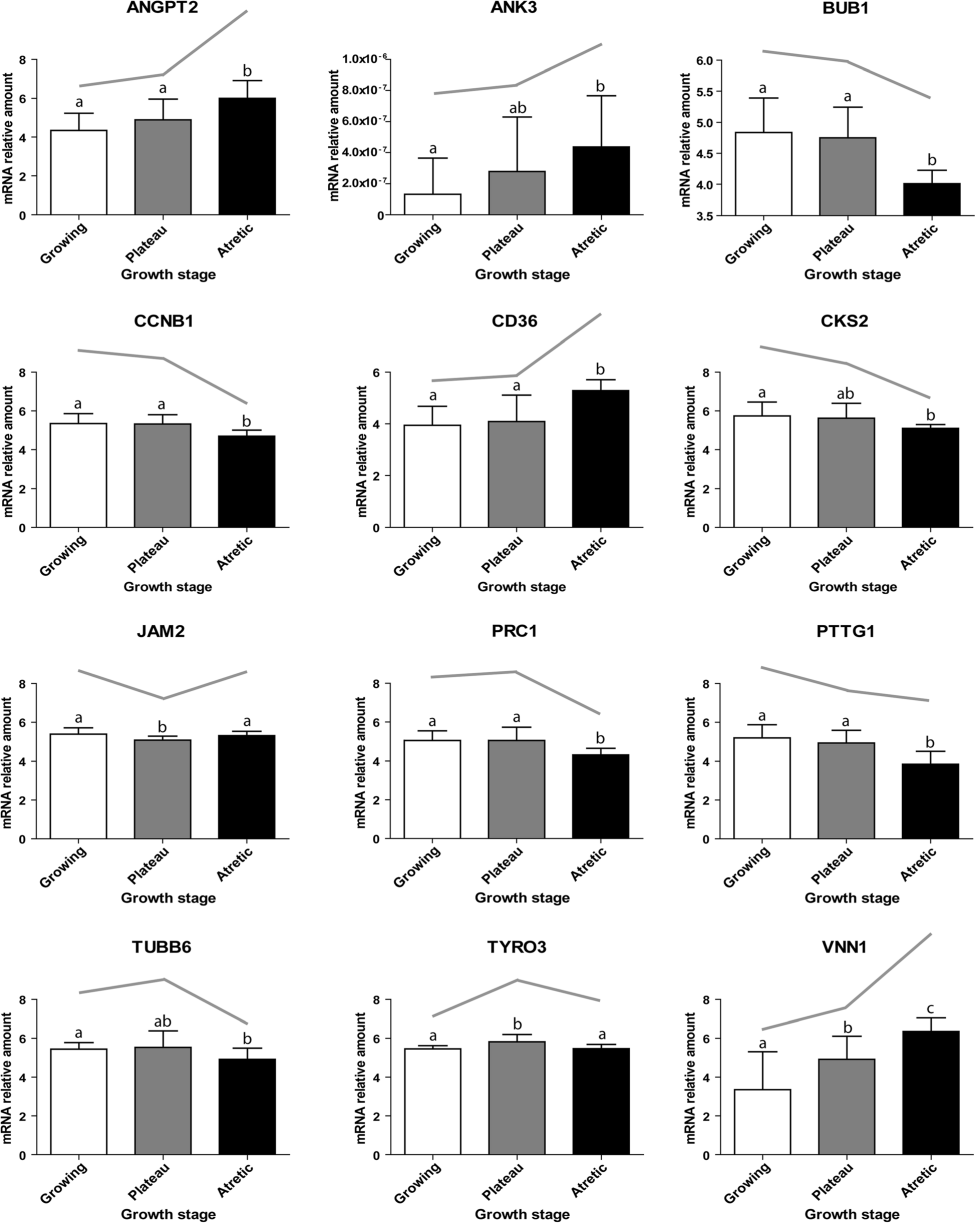
**Graphs of gene expression profiles showing a significant difference.** Twelve genes measured by qRT-PCR in the growing (white box; n = 7), plateau (grey box; n = 7) and atretic (black box; n = 6) follicles had significantly different expression levels (p-value < 0.05). Significantly different levels correspond to different letters above the bars. Error bars represent standard deviation (SD). The grey line above the boxes shows the corresponding microarray hybridization profiles. *ANGPT2*, Angiopoietin 2; *ANK3*, Ankyrin 3; *BUB1*, BUB1 mitotic checkpoint serine/threonine kinase; *CCNB1*, Cyclin B1; *CD36*, Cluster of Differentiation 36 (fatty acid translocase); *CKS2*, CDC28 protein kinase regulatory subunit 2; *JAM2*, Junction adhesion molecule 2; *PRC1*, Protein regulator of cytokinesis 1; *PTTG1*, Pituitary tumor-transforming 1; *TUBB6*, Tubulin, beta 6 class V; *TYRO3*, tyrosine-protein kinase receptor TYRO3; *VNN1*, vanin 1.

The selected genes were quantified in the four hybridized samples and two or three additional samples for each stage, and the results were compared to the differences observed with microarray analysis. One-tailed T tests revealed that among the 25 selected genes, three (*ACE2*, *NRP1* and *TRIB2*) had an expression level that was similar (p-value > 0.1) for all stages. For the G vs P contrast, three genes (*JAM2*, *TYRO3* and *VNN1*) had expression levels that were significantly different (p-value < 0.05) between the two stages, while the expression level of seven genes (*APOA1*, *RARRES1*, *STAR*, *BMP4*, *NR4A1*, *SERPINE* and *MT2A*) tended to be different (p-value < 0.1). For the P vs A contrast, nine genes (*ANGPT2*, *CCNB1*, *CD36*, *JAM2*, *PRC1*, *TYRO3*, *VNN1*, *PTTG1* and *BUB1*) had expression levels that were significantly different (p-value < 0.05) and four genes (*CKS2*, *TUBB6*, *NMB* and *BMP4*) had expression levels that tended to be different (p-value < 0.1). For the last contrast, G vs A, ten genes (*ANGPT2*, *ANK3*, *CCNB1*, *CD36*, *CKS2*, *PRC1*, *VNN1*, *PTTG1*, *TUBB6* and *BUB1*) had significantly different expression levels (p-value < 0.05) and three genes (*NMB*, *RARRES1*, *MT2A*) tended to have different (p-value < 0.01) expression profiles. Three genes (*ANGPT2*, *CD36* and *VNN1*) had an expression pattern that increased across the stages in a linear way (p-value < 0.05) and four genes (*CCNB1*, *PRC1*, *PTTG1* and *BUB1*) had an expression pattern that decreased in a linear way (p-value < 0.05). The expression level of the gene *RELN* was undetectable in four out of seven samples of the growing stage, while it was present in all the other samples. Transcripts for *ANKRD1* were found in five out of seven samples at the growing stage, in six out of seven at the plateau stage and in five out of six samples at the atretic stage. All the differences cited above were in accordance with the microarray profiles, but the levels of significance observed in the microarrays were not confirmed in the qRT-qPCR data due to the difference of power between the two methods with four and seven samples (Figure 3, Additional file 3: Figure S1 and Additional file 4: Figure S2).

### Functions

Using the datasets of modulated genes from the different contrasts, IPA Functions analysis generated statistically significant predictions for increased (z-score > 2) or decreased (z-score < −2) activity in several cellular functions. In the transition from G to P, 51 functions were predicted to be increased. Those functions were mostly related to cell death and survival, cellular movement, inflammatory response, metabolism and transport. The only decreased function in the contrast between G and P was related to cell morphology. For the contrast between P and A, 56 functions were predicted to be decreased. Among them were functions related to cardiovascular system development and function, cell death and survival, cellular assembly and organization, cell development, cellular movement and cell growth and proliferation. The 14 increased functions in the atretic phase in contrast to the plateau phase were related to cell death and survival, inflammatory response, metabolism and cell cycle (ploidy). (Additional file 5: Table S3).

### Upstream regulators

**Figure 4:**
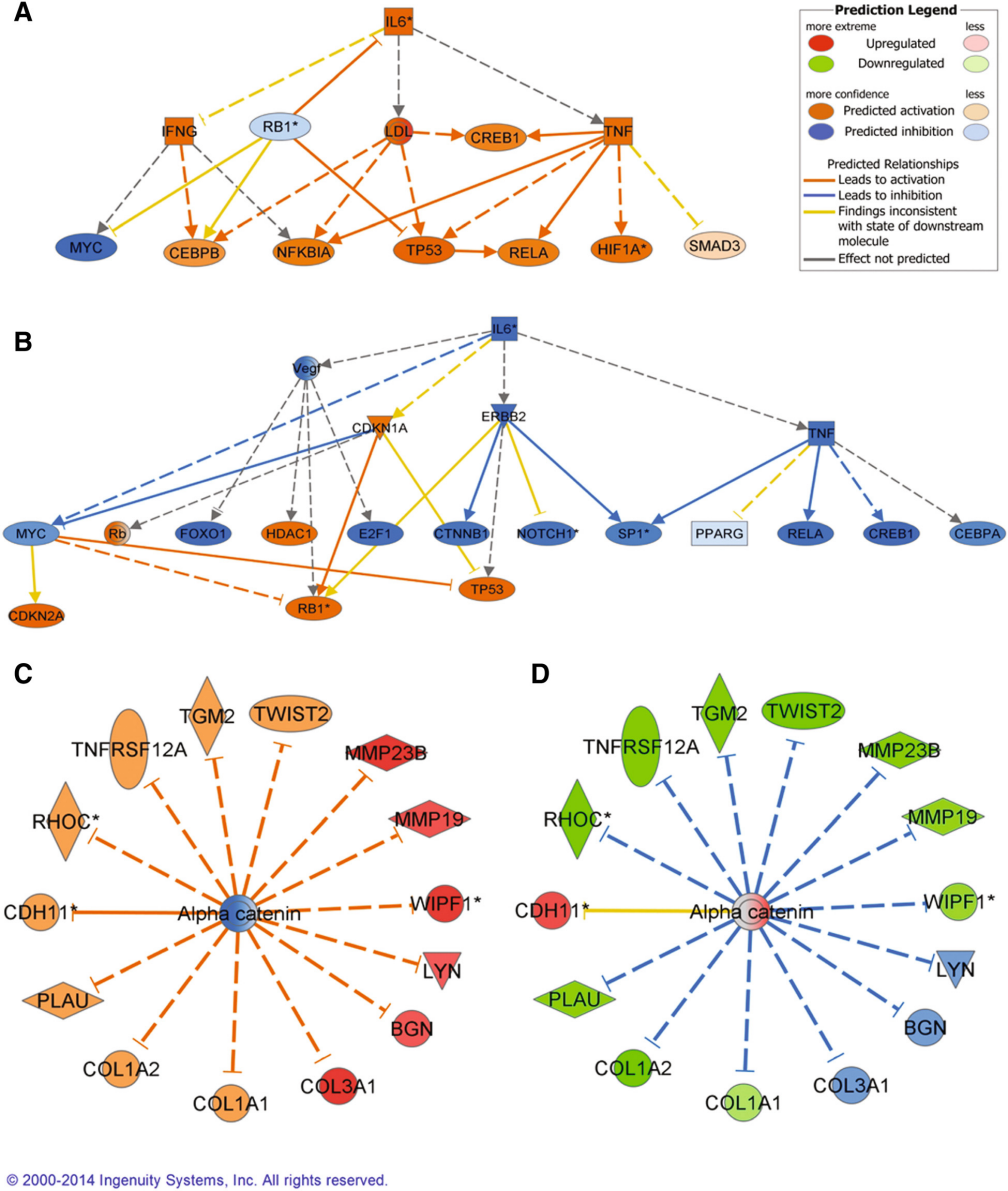
**Upstream regulators of interest.** The analysis of genes that are downstream of *IL-6* generated highly significant P values for this top regulator in the contrast Growing vs Plateau **(A)** and Plateau vs Atresia **(B)**. The same approach also generated highly significant P values for the top regulator Alpha catenin in the contrast Growing vs Plateau **(C)** and Plateau vs Atresia **(D)**. A list of abbreviations is provided at the end of the article.

For the G vs P contrast, significant activation (z-score > 2) of 23 upstream regulators was predicted. Those regulators included several chemical drugs, cytokines, growth factors and transcription factors. In the same contrast, five upstream regulators were predicted to be inhibited. Among them was a chemical product, enzymes and a transcription regulator. The change observed in the contrast between the plateau phase and the atretic phase was more drastic: 147 upstream regulators were predicted to be inhibited, while 61 were activated. The list of inhibited regulators included but was not limited to: chemical drugs or products, cytokines, enzymes, growth factors, kinases and transcription regulators. The list of activated upstream regulator for the P vs A contrast included chemical drugs or products, mature microRNAs and transcription regulators. IL-6 and Alpha catenin were two of the most relevant upstream regulators, as they were important in both contrasts (Figure 4). A selection of the upstream regulators with the strongest predictions is listed in Additional file 6: Table S4, and the most relevant will be discussed in a subsection in the discussion.

## Discussion

**Figure 5:**
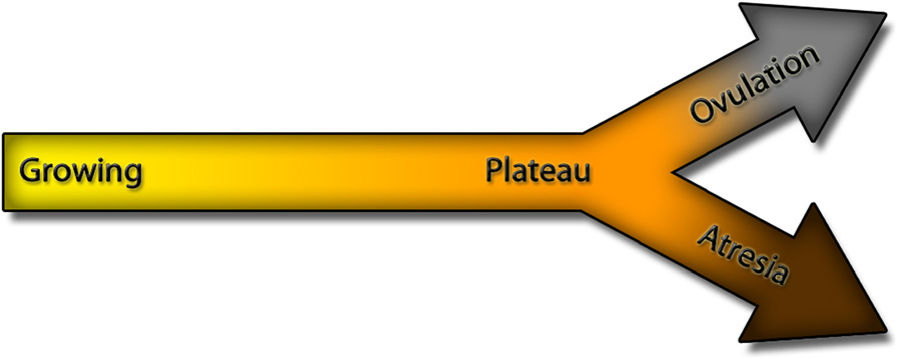
**Schematic representation of the follicle’s progression through the growth stages.** The dominant follicle undergoes three different growth stages during its development. The growing stage (yellow portion of the arrow) is characterised by a quick increase in diameter and the proliferation of granulosa cells. The mitoses slow down during the plateau phase (orange portion of the arrow), and the cells start a differentiation process. At this point, the follicle can take two different paths: progress toward the pre-ovulatory stage and ovulation (grey portion of the arrow) or become atretic (brown portion of the arrow), a stage characterised by an increase in apoptosis and cell death.

This study was the first to characterise transcriptomic profiles of granulosa cells obtained from follicles at different stages of dominance and obtained by objective flow cytometric analysis. The Between Group Analysis of the microarray data resulted in clear segregation of the three physiological states: growth, plateau and atresia which confirmed the value of the method developed by Blondin et al. [20] to identify follicular atresia. The segregation of the samples in three distinct clusters indicated that the stages are physiologically different, and the absence of a common axis between the three clusters supported the idea that the transition is not a linear process. As follicles go through the stages, some genes are activated while others are inactivated, instead of a continuous progression toward atresia. A similar segregation was observed in bovine follicles of 6 to 9 mm in diameter [19], supporting the assumption that the three different physiological states (growth, plateau and atresia) are present throughout the antral phase of folliculogenesis. The physiological difference between these growth stages was confirmed by the number of differentially expressed genes observed in each contrast. The small number of genes modulated in the same fashion across all the contrasts supported the above assumption that these changes are not linear, but rather really specific to a particular transformation during follicular development. The Figure 5 represents a simplified representation of the follicle progression through the growth stages.

The general analysis of gene functions (Additional file 5: Table S3) indicated that there was a clear rise of cell death signalling and apoptosis as early as the plateau phase, and that a decrease of cell viability took place between the plateau and the atretic stage. This function was associated with the modulation of several genes in the same direction, as shown by the z-scores above 2. Simultaneously, inflammation signalling started to increase in the plateau phase along with protein synthesis. This represented a transition from cell division to differentiation, characterized by increased lipid metabolism and steroid synthesis typical of dominant follicles. In the next transition, from plateau to atresia, many of the genes being shut down were associated with cancer, cell viability and angiogenesis, illustrating the growth arrest and non-reversible state of these follicles. The few functions that were activated in atresia compared to plateau were cell death-related. In this case, analysis of individual DEG is a good way to identify early and late markers of atresia.

### Upstream regulators

One of the most powerful tools in Ingenuity Pathway Analysis is the identification of upstream regulators. The software categorizes upregulated or downregulated genes that are in relation with a specific trigger, such as a gene or a chemical. For example, if tens of genes that normally respond positively or negatively to estradiol are all moving in the anticipated direction, this gives a strong support to a real physiological phenomenon. Additional file 6: Table S4 documents the importance of certain immunomodulators (*IL-1, Il-6* and *TNF*) in the differentiation process [39-41] expected in the plateau phase. In our data set, numerous genes modulated by *IL-6* underwent opposite changes as the follicles went from growing to plateau and then from plateau to atresia (Figure 4 A and B), supporting the idea that the physiology of the plateau phase is different from the other stages. This molecule plays a role in inflammation [42] and could potentially be used as an indicator to predict the general fate of the follicles. The combination of two different *TGFB* pathways also supports the shift from growth to differentiation, as these molecules seem to be important factors for tissue remodelling during wound healing [43] and are known to participate in the differentiation of granulosa cells [44]. On the other hand, the decrease in expression of *c-Myc* targets supports the decrease in cell divisions in this class. In myeloid leukemia cells, the decrease of *c-Myc* has been linked with differentiation [45].

Some upstream regulators suggested for the transition between the plateau and the atretic phase moved in the opposite direction of what would be expected in pre-ovulatory follicles. For example, *TGFB1* was predicted to be downregulated in our atretic follicles; however, it has been suggested to participate in luteal function [46] and to have an anti-apoptotic effect in human luteinized granulosa cells [47].

Alpha catenin is a key element of the cadherin-catenin protein complex which participates in intercellular adhesion and plays a pivotal role in development [48]. In epithelial cells, alpha catenin is a mediator of cell density [49] and it has been postulated that it could be part of a molecular biosensor of cell density and positioning [48]. Over-expression of this gene has been associated with increased osteoblastic differentiation [50] while reduced expression was found in highly proliferative and poorly differentiated lung cancer tumour cells [51]. Administration of hCG–LH in rats reduced alpha catenin expression in ovulatory follicles and the authors suggested that it plays a role in the changes in cell-cell adhesion associated with ovulation and luteinisation [52]. Our results support this idea, as the downregulation of alpha catenin was predicted to be one of the upstream regulators of the changes in gene expression between the growing and the plateau phase. Follicles at the plateau phase undergo changes that prepare them for an eventual ovulation, and alpha catenin may play a role in this process. On the other hand, the activation of the same molecule was proposed as an upstream regulator of the transition from plateau to atretic follicles. This view is supported by data from Sanchez et al. who suggested that beta-catenin, which is closely related to alpha catenin, is involved in granulosa cell atresia [53]. The shift observed in the action of genes regulated by alpha catenin as follicles go from growing to plateau and from plateau to atresia (Figure 4C and D) suggests that this molecule acts as an initiator of some of the changes occurring during folliculogenesis.

Hepatic growth factor (*HGF*) has been associated with increased granulosa cell proliferation in mouse follicles [54]. It also stimulates bovine granulosa cell proliferation *in vitro* [55] and has anti-apoptotic effects on rat granulosa cells *in vitro* [56]. It has been suggested that *HGF* controls theca cell and granulosa cell steroidogenesis [57], indicating an important role during folliculogenesis. *HGF* is an upstream regulator predicted to be downregulated in our contrast P vs A, which is consistent with the proliferative and anti-apoptotic effect of that molecule on granulosa cells. The transition from plateau to atretic follicles includes an increase in apoptotic events in granulosa cells and *HGF* may play a role in this process.

### Biomarkers

With the important place taken by reproduction management in cattle, accessible tools to identify the growth stages of the follicles would be useful in many situations. As the physiology and the differentiation of the follicle can have an important impact on the competence of the oocyte within [17], the classification of follicles among the different growth stages may improve the programs currently used for cattle reproductive management. For example, Ovsynch is an effective program used to synchronise ovulation in lactating dairy cows, but it is less effective with heifers [58]. Even among lactating cows, different pregnancy rates are observed between primiparous and multiparous cows [59]. Those different success rates could be caused by small differences in follicular development, which are impossible to detect with current methods. In humans, numerous studies tried to find a good predictor of oocyte competence in follicular fluid, but none has yet succeeded (see [60] for a review), and in cattle, follicular diameter is not always linked with competence [61]. The results of this project suggest a list of atresia markers which can be tested in larger follicles, in order to identify atretic follicles containing poor quality oocyte.

Several biomarker candidates showed no significant differences between the stages. However, the absence of significance was often caused by an important standard deviation in the plateau group. The standard deviation of seven genes (*RARRES1*, *SERPINE1*, *STAR*, *TRIB2*, *CYP19A1*, *APOA1*, *BMP4*), from which several are linked to LH response [34,62,63] or to progesterone [64,65], was higher in the plateau group than in the two others. This may be an indicator of a more diversified population of follicles. This assumption would be coherent with our understanding of this particular stage, as we supposed that the follicles from the plateau phase can take two directions: proceed toward ovulation or enter atresia. The physiological differences between those two options may explain the important variation observed among the samples of the plateau phase.

### Markers of growing follicles

Growing follicles shared many characteristics with the plateau phase, while some genes had a clear pattern of change when follicles were moving into atresia. For example, transcripts for *RELN* were not found at all (with PCR) in four out of seven granulosa cell samples originating from follicles at the growing stage, while they were found in all the samples from the other categories in similar quantities (Additional file 4: Figure S2). RELN is a ligand of the very-low-density lipoprotein receptor (VLDLR) and has an antiproliferative activity [66]. This gene had already been associated with the end of follicular growth [67] and could be used as a marker.

### Markers of the plateau phase

Among the tested genes, only a few had an expression level that was significantly different between the plateau phase and other phases. Those genes were *TYRO3* and *JAM2*, which were upregulated and downregulated, respectively. TYRO3 is a receptor of GAS6 (growth arrest specific 6), and both the receptor and the ligand, were abundantly expressed in differentiating stem cells [68]. The over expression of the receptor TYRO3 in samples from the plateau phase is another element that highlights the importance of the differentiation process at that stage of folliculogenesis. *JAMs* are a family of genes that encode proteins that are localized at the junctions between cells. They have multiple functions including angiogenesis, cell migration and proliferation [69]. Blocking *JAM2* and *JAM3 in vitro* impaired the growth and invasion of glioma cells [70], and their expression was increased in tumor cells [71]. The downregulation of *JAM2* observed in the plateau phase is consistent with the slowing of proliferation. Thus, the expression patterns of *TYRO3* and *JAM2* in this study support the idea that the plateau phase is physiologically distinct from the growing and the atretic phases.

### Markers of follicular atresia

Several genes were differentially expressed between the atretic phase and the other phases making them potential biomarkers. Among them are genes related to angiogenesis such as *ANGPT2* and *CD36*, which were over expressed in the atretic group. The status of blood vessel network is important during folliculogenesis, as an increased vascularisation is linked with better delivery of gonadotropins [72] and thecal blood vessel regression is linked with atresia of bovine non-ovulatory follicles [73]. Both *ANGPT2* and *CD36* can have anti-angiogenic effects [74,75]. *ANK3* expression was also significantly higher at the atretic phase compared to the other phases. This protein participates in the coordination of cell membrane assembly by interacting with E-cadherin at contact sites between cells [76] and it has also been suggested as a marker of senescence in cultured fibroblast cells [77].

A decreased expression of genes related to cell proliferation (*BUB1*, *CCNB1*, *CKS2*, *TUBB6* and *PRC1*) was also observed in atretic follicles. *BUB1* is a key regulator of spindle assembly checkpoint and chromosomal segregation during mitosis and meiosis [78]; *CCNB1* encodes for a protein that is essential for cells to enter mitosis [79]; *CKS2* has been suggested to serve as a cell cycle checkpoint protein for the S/G2 transition [80]; *TUBB6* is a minor isoform of tubulin that is essential for cell cycle [81]; *PRC1* helps to control proliferation by regulating DNA replication [82]. The sharp decrease in the expression of these genes observed in atretic follicles is consistent with the reduced proliferation that characterises this stage, and suggests that they can be used as biomarkers to identify follicles committed to atresia.

The linear increase in the expression of *VNN1* across all stages was consistent with the literature, as this transcript increased during the coasting period in follicles [67,83] and had been linked to follicular atresia. VNN1 is an enzyme that regulates the GSH-dependent response to oxidative injury, decreases tissue resistance to oxidative stress [84], and its over expression was consistent with the increase in apoptosis observed during follicular atresia. The progression toward the atretic stage was also accompanied by a linear decrease of *PTTG1*, whose suppression has been linked to increased apoptosis [85]. When over expressed, this gene limited the ability of p53 to induce apoptosis [86].

## Conclusion

In conclusion, this project provided valuable clues to the physiological differences between the three growth stages of dominant follicles. It led to the identification of upstream regulators associated with functional changes that take place during the late development of follicles such as alpha catenin, *HGF*, *IL-6* and *TGFBs*. It also shed light on the principal functions modulated during those changes and finally, it provided numerous potential biomarkers to simplify future determination of the growth stage of follicles.

## Additional files

Additional file 1: Table S1. qRT-PCR primer sequences, product size, annealing temperature and accession number for the studied genes. Additional file 2: Table S2. Gene names of qRT-PCR validation candidates. Additional file 3: Figure S1. Graphs of gene expression profiles showing no significant difference. Additional file 4: Figure S2. Graphs of gene transcripts presence in the different growth phases. Additional file 5: Table S3. Functions analysis in Ingenuity Pathway Analysis (IPA) software for the G vs P contrast (A) and the P vs. A contrast (B). Additional file 6: Table S4. Upstream analysis in Ingenuity Pathway Analysis (IPA) software for the Growing vs. Plateau (A) and the Plateau vs Atretic (B) contrasts.

## Abbreviations

A: Atretic
ACE2: Angiotensin I converting enzyme 2
ANGPT2: Angiopoietin 2
ANK3: Ankyrin 3
ANKRD1: Ankyrin repeat domain 1
ANOVA: Analysis of variance
APOA1: Apolipoprotein A-I
ART: Assisted reproductive technologies
BGA: Between group analysis
*BGN*: Biglycan
BMP4: Bone morphogenetic protein 4
BUB1: Budding uninhibited by benzimidazoles 1 (BUB1 mitotic checkpoint serine/threonine kinase)
CCNB1: Cyclin B1
CD36: Cluster of Differentiation 36 (fatty acid translocase)
*CDH11*: Cadherin 11
*CDKN1A*: Cyclin-dependent kinase inhibitor 1A
*CDKN2A*: Cyclin-dependant kinase inhibitor
cDNA: Complementary DNA
*CEBPA*: CCAAT/enhancer binding protein(C/EBP)alpha
*CEBPB*: CCAAT/enhancer binding protein(C/EBP) beta
CKS2: CDC28 protein kinase regulatory subunit 2
*COL1A1*: Collagen type I alpha 1
*COL1A2*: Collagen type 1 alpha 2
*COL3A1*: Collagen type III alpha 1
*CREB1*: cAMP responsive element binding protein 1
*CTNNB1*: Catenin cadherin-associated protein beta 1
CYP17A1: Cytochrome P450, family 17, subfamily A, polypeptide 1
CYP19A1: Cytochrome P450, family 19, subfamily A, polypeptide 1
DEG: Differentially expressed genes
*E2F1*: E2F transcription factor 1
EDTA: Ethylenediaminetetraacetic acid
EIF2B2: Eukaryotic translation initiation factor 2B, subunit 2 beta
*ERBB2*: V-erb-b2 avian erythroblastic leukemia viral oncogene homolog 2
EtOH: Ethanol
*FOXO1*: Forkhead box O1
FSC: Forward light scatter
FSH: Follicle-stimulating hormone
G: Growing
GAS6: Growth arrest specific 6
hCG: Human chorionic gonadotropin
*HDAC1*: Histone deacetylase 1
HGF: Hepatic growth factor
*HIF1A*: Hypoxia inducible factor subunit alpha
*IFNG*: Interferon gamma
IL-1: Interleukin 1
*IL6*: Interleukin 6
IPA: Ingenuity pathway analysis
IVT: In vitro transcription
JAM2: Junction adhesion molecule 2
JAM3: Junctional adhesion molecule C
*LDL*: Low density lipoprotein
LH: Lluteinizing hormone
*LYN*: V-yes-1 Yamaguchi sarcoma viral related oncogene homolog
*MMP19*: Matrix metallopeptidase 19
*MMP23B*: Matrix metallopeptidase 23B
MT2A: Metallothionein 2A
*MYC*: V-myc myelocytomatosis viral oncogene homolog
*NFKBIA*: Nuclear factor of kappa light polypeptide gene enhancer in B-cells inhibitor alpha
NMB: Neuromedin B
*NOTCH1*: Notch 1
NR4A1,Nuclear receptor subfamily 4: group A, member 1
NRP1: Neuropilin 1
P: Plateau
P53: Tumor suppressor p53
PBS: Phosphate buffered saline
PBSE: PBS-EDTA solution
PI: Propidium iodide
*PLAU*: Plasminogen activator urokinase
*PPARG*: Peroxisome proliferator-activated receptor gamma
PRC1: Protein regulator of cytokinesis 1
PTTG1: Pituitary tumor-transforming 1
qRT-PCR: Quantitative real-time PCR
RARRES1: Retinoic acid receptor responder (tazarotene induced) 1
*Rb*: Rb tumor suppressor
*RB1*: Retinoblastoma 1
RELA: V-rel avian reticuloendotheliosis viral oncogene homolog A
RELN: Reelin
*RHOC*: Ras homolog family member C
RIN: RNA integrity number
SD: Standard deviation
SERPINE1: Serpin peptidase inhibitor, clade E (nexin, plasminogen activator inhibitor type 1), member 1
SF3A1: Splicing factor 3a, subunit 1
*SMAD3*: SMAD family member 3
*SP1*: Sp1 transcription factor
STAR: Steroidogenic acute regulatory protein
TGFB: Transforming growth factor beta
*TGM2*: Transglutaminase 2
*TNF*: Tumor necrosis factor
*TNFRSF12A*: Tumor necrosis factor receptor superfamily member 12A
*TP53*: Tumor protein p53
TRIB2: Tribbles pseudokinase 2
TUBB6: Tubulin, beta 6 class V
*TWIST2*: Twist basic helix-loop-helix transcription factor 2
TYRO3: Tyrosine-protein kinase receptor TYRO3
*Vegf*: Vascular endothelial growth factor A
VLDLR: Very-low-density lipoprotein receptor
VNN1: Vanin 1
*WIPF1*: WAS/WASL interacting protein family member 1
