# Supplementary material for: Global gene expression in granulosa cells of growing, plateau and atretic dominant follicles in cattle
Source: Reprod Biol Endocrinol. 2015 Mar 8;13:17. doi: 10.1186/s12958-015-0010-7 (PMC4355352; doi:10.1186/s12958-015-0010-7)
Supplement: Additional file 1: Table S1. — qRT-PCR primer sequences, product size, annealing temperature and accession number for the studied genes. [file 12958_2015_10_MOESM1_ESM.doc]

**Additional file 1. qRT-PCR primer sequences, product size, annealing temperature and accession number for the studied genes.**

| **Gene Symbol** | **Primer sequence** | | **Product Size (bp)** | **Annealing Temperature (°C)** | **GenBank Accession Number** |
| --- | --- | --- | --- | --- | --- |
| ACE2 | fwd: | GAGCAAGTGTGTGGAATCTG | 303 | 53 | XM_005228429.1 |
|  | rev: | GTGATATTGTCAGAAAGACGGT |  |  |  |
| ANGPT2 | fwd: | GAAAGCCTTCGGGAAGATAAA | 416 | 53 | NM_001098855.1 |
|  | rev: | ACTAAGCTGTCAATACACACG |  |  |  |
| ANK3 | fwd: | GGGTGCTCTTAGTGCTTAAA | 222 | 53 | NM_001105620.1 |
|  | rev: | CTAGTATGACATGGCGAGAATG |  |  |  |
| ANKRD1 | fwd: | AATGAGGTTTGGAGCACAGGGA | 487 | 59 | NM_001034378.2 |
|  | rev: | TGGCCCATGTTCCTTCCACTTA |  |  |  |
| APOA1 | fwd: | CCGTGTATGTGGAAGCAATCAAGG | 100 | 59 | NM_174242.3 |
|  | rev: | GTTGTCCAGGAGTTTCAGGTTGAG |  |  |  |
| BMP4 | fwd: | GCTGGAATGACTGGATTGT | 229 | 53 | NM_001045877.1 |
|  | rev: | CTCCACTACCATCTCCTGATA |  |  |  |
| BUB1 | fwd: | CAGGCTAATTGTACTGCTCTT | 276 | 54 | NM_001102011.2 |
|  | rev: | TGTCCTCATAAATGCTTGTATCC |  |  |  |
| CCNB1 | fwd: | GGAACTAACTATGCTGGACTAC | 268 | 53 | NM_001045872.1 |
|  | rev: | GTGCTAGAGTGCTGATCTTAG |  |  |  |
| CD36 | fwd: | CAGAGAGATGGTCATTCTACAC | 250 | 53 | NM_001278621.1 |
|  | rev: | TGCTGATGCTGTACAAAGG |  |  |  |
| CKS2 | fwd: | TTCGATGAACACTACGAATACC | 330 | 54 | NM_001113319.2 |
|  | rev: | GAATACAGCTCATGCACAGG |  |  |  |
| CYP17A1 | fwd: | ACCATCAGAGAAGTGCTCCGAA | 114 | 59 | NM_174304.2 |
|  | rev: | CCACAACGTCTGTGCCTTTGT |  |  |  |
| CYP19A1 | fwd: | GCGTTGTCTAAGCAAACTCTCCCA | 462 | 59 | NM_174305.1 |
|  | rev: | CAGCTTCCAACTGGCATTTCCCAT |  |  |  |
| EIF2B2 | fwd: | GAACTCATACTCTAGCACTGG | 262 | 53 | NM_001015593.1 |
|  | rev: | GTAGATGTAGGAAGGTGCATT |  |  |  |
| JAM2 | fwd: | ATCAGCTACATGCACCCTCTGT | 173 | 59 | NM_001083736.1 |
|  | rev: | ATTCTGCCACCGTTCTGTGACT |  |  |  |
| MT2A | fwd: | ATCCTTTGCTCAGCAGTCTC | 290 | 54 | NM_001075140.1 |
|  | rev: | ATATGCAGGTTTGTACACGTTG |  |  |  |
| NMB | fwd: | CGGTCACTTCATGGGCAA | 260 | 54 | NM_001075270.2 |
|  | rev: | TGGGTGGGCACAATTTCA |  |  |  |
| NR4A1 | fwd: | CAGCACTTCTAAACTGGACTAC | 216 | 54 | NM_001075911.1 |
|  | rev: | GAGGATAAAGAGCTCCAGAAAG |  |  |  |
| NRP1 | fwd: | AAGGCAAGGGCTCCGAAGATTT | 251 | 59 | NM_001205660.1 |
|  | rev: | TTGTTTGCCATTCCCAGCAGGT |  |  |  |
| PRC1 | fwd: | CACACATGCTTGTCTGAACT | 330 | 54 | NM_001076543.2 |
|  | rev: | GACCAAATCCACACCTGTAAG |  |  |  |
| PTTG1 | fwd: | CAGAAACAGACAACTTTCTCTACC | 321 | 54 | NM_001034310.2 |
|  | rev: | CCAGAGTAGACAGAATGCTTGAG |  |  |  |
| RARRES1 | fwd: | CAGAAGTCAACACCGTTTCTA | 270 | 53 | NM_001075430.2 |
|  | rev: | AGAAATTACTAAGCTCCGTCAG |  |  |  |
| RELN | fwd: | TCCTGCGGGTCATATTCACACCTT | 124 | 59 | NM_001206458.1 |
|  | rev: | AGCTTGGTTCACCCTAAAGTGC |  |  |  |
| SERPINE1 | fwd: | ACCATCCAACTTCGGCTCACTT | 494 | 54 | NM_174137.2 |
|  | rev: | TACTGAGTGTGGCTGTCACTGT |  |  |  |
| SF3A1 | fwd: | TGTGTCCCTCTTGCTGAGTT | 210 | 59 | NM_001081510.1 |
|  | rev: | ATCGCATCCTACAGGGCATT |  |  |  |
| STAR | fwd: | GAATGCGGACAAGGCTCTTCTAAC | 407 | 57 | NM_174189.2 |
|  | rev: | GACTCCTGTCATGGAAGAAGTACG |  |  |  |
| TRIB2 | fwd: | CACACATCTTGGCATCGCACTGTT | 182 | 59 | NM_178317.3 |
|  | rev: | AGCACCCAGGTTTCACATCAGTCT |  |  |  |
| TUBB6 | fwd: | GCCATCCAGAACAAGAACAG | 440 | 54 | NM_001046373.1 |
|  | rev: | CACACTTAGACGGGACTCTC |  |  |  |
| TYRO3 | fwd: | CTGAGCATGCTACCATATCC | 283 | 53 | NM_001191228.1 |
|  | rev: | TAAACAGCATCTAACGGCTT |  |  |  |
| VNN1 | fwd: | TGGCACGTTTGGAACCCAGTAT | 222 | 59 | NM_001024556.2 |
|  | rev: | CTTTGGATTGAGCCTTAGCGCTTG |  |  |  |
