# Supplementary material for: Global gene expression in granulosa cells of growing, plateau and atretic dominant follicles in cattle
Source: Reprod Biol Endocrinol. 2015 Mar 8;13:17. doi: 10.1186/s12958-015-0010-7 (PMC4355352; doi:10.1186/s12958-015-0010-7)
Supplement: Additional file 2: Table S2. — Gene names of qRT-PCR validation candidates. [file 12958_2015_10_MOESM2_ESM.doc]

**Additional file 2. Gene names of qRT-PCR validation candidates**.

| **Gene Symbol** | **Gene Name** |
| --- | --- |
| ACE2 | Angiotensin I converting enzyme 2 |
| ANGPT2 | Angiopoietin 2 |
| ANK3 | Ankyrin 3 (ankyrin G) |
| ANKRD1 | Ankyrin repeat domain 1 |
| APOA1 | Apolipoprotein A-I |
| BMP4 | Bone morphogenetic protein 4 |
| BUB1 | Budding uninhibited by benzimidazoles 1 (BUB1 mitotic checkpoint serine/threonine kinase) |
| CCNB1 | Cyclin B1 |
| CD36 | Cluster of Differentiation 36 (fatty acid translocase) |
| CKS2 | CDC28 protein kinase regulatory subunit 2 |
| CYP17A1 | Cytochrome P450, family 17, subfamily A, polypeptide 1 |
| CYP19A1 | Cytochrome P450, family 19, subfamily A, polypeptide 1 |
| JAM2 | Junction adhesion molecule 2 |
| MT2A | Metallothionein 2A |
| NMB | Neuromedin B |
| NR4A1 | Nuclear receptor subfamily 4, group A, member 1 |
| NRP1 | Neuropilin 1 |
| PRC1 | Protein regulator of cytokinesis 1 |
| PTTG1 | Pituitary tumor-transforming 1 |
| RARRES1 | Retinoic acid receptor responder (tazarotene induced) 1 |
| RELN | Reelin |
| SERPINE1 | Serpin peptidase inhibitor, clade E (nexin, plasminogen activator inhibitor type 1), member 1 |
| STAR | Steroidogenic acute regulatory protein |
| TRIB2 | Tribbles pseudokinase 2 |
| TUBB6 | Tubulin, beta 6 class V |
| TYRO3 | Tyrosine-protein kinase receptor TYRO3 |
| VNN1 | Vanin 1 |
