# Supplementary material for: Global gene expression in granulosa cells of growing, plateau and atretic dominant follicles in cattle
Source: Reprod Biol Endocrinol. 2015 Mar 8;13:17. doi: 10.1186/s12958-015-0010-7 (PMC4355352; doi:10.1186/s12958-015-0010-7)

Additional file 3: Graphs of gene expression profiles showing no significant difference.

Fifteen genes measured by qRT-PCR in the growing (white box; n = 7), plateau (grey box; n = 7) and atretic (black box; n = 6) follicles had no significantly different expression levels (P > 0.05). Significantly different levels correspond to different letters above the bars. Error bars represent standard deviation (SD). *ACE2*, Angiotensin I converting enzyme 2; *APOA1*, Apolipoprotein A-I; *BMP4*, Bone morphogenetic protein 4; *CYP17A1*, Cytochrome P450, family 17, subfamily A, polypeptide 1; *CYP19A1*, Cytochrome P450, family 19, subfamily A, polypeptide 1; *MT2A*, Metallothionein 2A; *NMB*, Neuromedin B; *NR4A1*, Nuclear receptor subfamily 4, group A, member 1; *NRP1*, Neuropilin 1; *RARRES1*, Retinoic acid receptor responder (tazarotene induced) 1; *SERPINE1*, Serpin peptidase inhibitor, clade E (nexin, plasminogen activator inhibitor type 1), member 1; *STAR*, Steroidogenic acute regulatory protein; *TRIB2*, Tribbles pseudokinase 2.

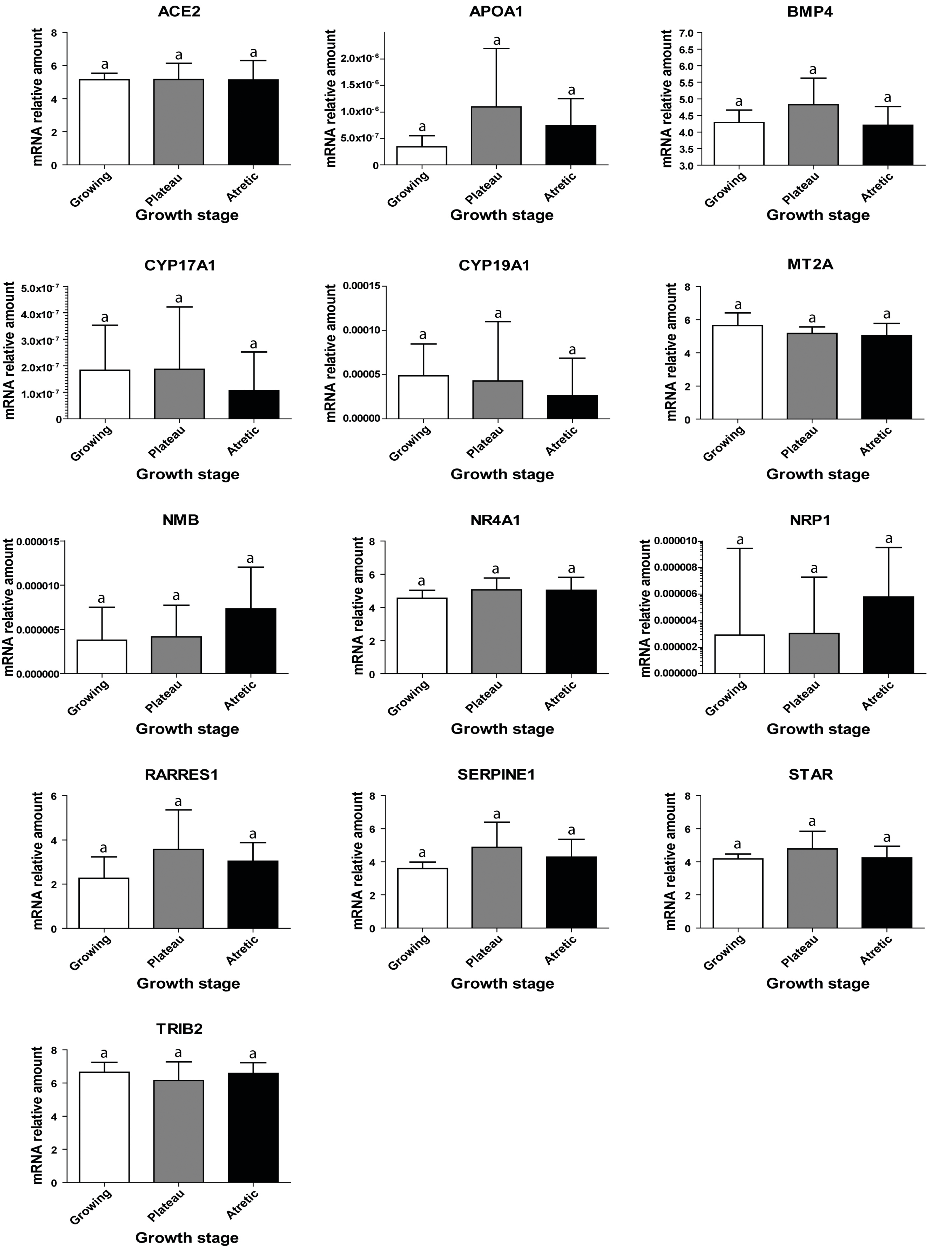

Supplement: Additional file 3: Figure S1. — Graphs of gene expression profiles showing no significant difference. [file 12958_2015_10_MOESM3_ESM.pdf]
