# Supplementary material for: Global gene expression in granulosa cells of growing, plateau and atretic dominant follicles in cattle
Source: Reprod Biol Endocrinol. 2015 Mar 8;13:17. doi: 10.1186/s12958-015-0010-7 (PMC4355352; doi:10.1186/s12958-015-0010-7)

Additional file 4: Graphs of gene transcripts presence in the different growth phases.
The transcripts of two genes measured by qRT-PCR in the growing (n = 7), plateau (n = 7) and atretic (n = 6) follicles were absent in several samples. The black boxes show the percentage of samples were the transcripts were found. ANKRD1, Ankyrin repeat domain 1 ; RELN, reelin.

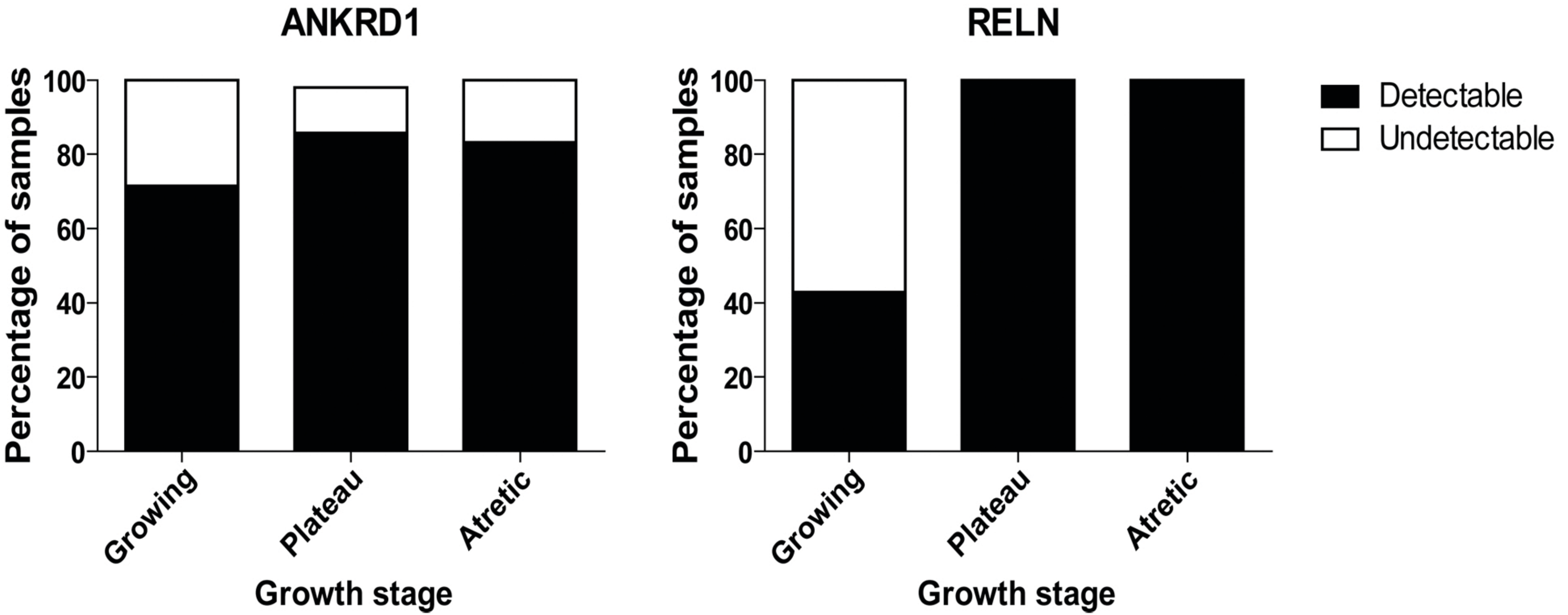

Supplement: Additional file 4: Figure S2. — Graphs of gene transcripts presence in the different growth phases. [file 12958_2015_10_MOESM4_ESM.pdf]
