# Supplementary material for: Global gene expression in granulosa cells of growing, plateau and atretic dominant follicles in cattle
Source: Reprod Biol Endocrinol. 2015 Mar 8;13:17. doi: 10.1186/s12958-015-0010-7 (PMC4355352; doi:10.1186/s12958-015-0010-7)
Supplement: Additional file 5: Table S3. — Functions analysis in Ingenuity Pathway Analysis (IPA) software for the G vs P contrast (A) and the P vs. A contrast (B). [file 12958_2015_10_MOESM5_ESM.doc]

**Additional file 5. Functions analysis in Ingenuity Pathway Analysis (IPA) software for the G vs P contrast (A) and the P vs. A contrast (B).** Only functions annotations that obtained a Regulation z-score value higher than the absolute value of 2, which is considered significant and therefore for which IPA could predict the Activation State, are presented. P-value < 0.05 calculated by Fisher’s Exact test; # Molecules = number of molecules associated to annotation in user’s dataset.

1. Functions – Growing vs Plateau contrast

| **Category** | **Diseases or Functions Annotation** | **p-Value** | **Predicted Activation State** | **Activation z-score** | **# Molecules** |
| --- | --- | --- | --- | --- | --- |
| Cell Death and Survival | cell death of fibroblasts | 2.56E-04 | Increased | 3,088 | 16 |
| Cell Death and Survival | apoptosis of fibroblasts | 6.51E-04 | Increased | 2,727 | 13 |
| Cell Death and Survival | apoptosis of tumor cell lines | 1.48E-05 | Increased | 2,681 | 53 |
| Cell Death and Survival | apoptosis of connective tissue cells | 2.66E-03 | Increased | 2,66 | 15 |
| Cell Death and Survival | cell death of tumor cell lines | 1.17E-04 | Increased | 2,472 | 58 |
| Cell Death and Survival | cell death of immune cells | 4.05E-03 | Increased | 2,461 | 28 |
| Cell Death and Survival | cell death | 5.36E-06 | Increased | 2,294 | 124 |
| Cell Death and Survival | cell death of connective tissue cells | 2.37E-05 | Increased | 2,284 | 33 |
| Cell Death and Survival | apoptosis | 6.37E-06 | Increased | 2,235 | 104 |
| Cell Death and Survival | necrosis | 5.74E-06 | Increased | 2,203 | 99 |
| Cell Death and Survival | cell death of blood cells | 3.92E-03 | Increased | 2,2 | 29 |
| Cell Death and Survival | cell death of myeloma cell lines | 6.62E-03 | Increased | 2,183 | 6 |
| Cell Death and Survival | cell death of macrophages | 1.26E-02 | Increased | 2,175 | 7 |
| Cell-To-Cell Signaling and Interaction | immune response of antigen presenting cells | 9.01E-03 | Increased | 2,03 | 9 |
| Cellular Compromise | degranulation of mast cells | 4.37E-03 | Increased | 2,26 | 8 |
| Cellular Compromise | stress response of cells | 9.23E-03 | Increased | 2,207 | 7 |
| Cellular Function and Maintenance | engulfment of cells | 5.97E-03 | Increased | 3,39 | 16 |
| Cellular Function and Maintenance | endocytosis | 2.58E-03 | Increased | 2,764 | 14 |
| Cellular Movement | cell movement of phagocytes | 1.21E-02 | Increased | 3,359 | 22 |
| Cellular Movement | migration of antigen presenting cells | 9.73E-04 | Increased | 3,112 | 11 |
| Cellular Movement | cell movement of antigen presenting cells | 1.06E-02 | Increased | 2,872 | 15 |
| Cellular Movement | migration of macrophages | 1.32E-03 | Increased | 2,599 | 7 |
| Cellular Movement | migration of cells | 1.81E-04 | Increased | 2,329 | 70 |
| Cellular Movement | cell movement | 4.45E-05 | Increased | 2,106 | 79 |
| Hematological System Development and Function | cell movement of phagocytes | 1.21E-02 | Increased | 3,359 | 22 |
| Hematological System Development and Function | migration of antigen presenting cells | 9.73E-04 | Increased | 3,112 | 11 |
| Hematological System Development and Function | cell movement of antigen presenting cells | 1.06E-02 | Increased | 2,872 | 15 |
| Hematological System Development and Function | migration of macrophages | 1.32E-03 | Increased | 2,599 | 7 |
| Hypersensitivity Response | degranulation of mast cells | 4.37E-03 | Increased | 2,26 | 8 |
| Immune Cell Trafficking | cell movement of phagocytes | 1.21E-02 | Increased | 3,359 | 22 |
| Immune Cell Trafficking | migration of antigen presenting cells | 9.73E-04 | Increased | 3,112 | 11 |
| Immune Cell Trafficking | cell movement of antigen presenting cells | 1.06E-02 | Increased | 2,872 | 15 |
| Immune Cell Trafficking | migration of macrophages | 1.32E-03 | Increased | 2,599 | 7 |
| Inflammatory Response | cell movement of phagocytes | 1.21E-02 | Increased | 3,359 | 22 |
| Inflammatory Response | immune response of cells | 7.17E-03 | Increased | 2,914 | 15 |
| Inflammatory Response | migration of macrophages | 1.32E-03 | Increased | 2,599 | 7 |
| Inflammatory Response | degranulation of mast cells | 4.37E-03 | Increased | 2,26 | 8 |
| Inflammatory Response | immune response of antigen presenting cells | 9.01E-03 | Increased | 2,03 | 9 |
| Lipid Metabolism | fatty acid metabolism | 2.50E-03 | Increased | 2,567 | 25 |
| Lipid Metabolism | secretion of steroid | 8.81E-03 | Increased | 2,183 | 7 |
| Lipid Metabolism | transport of lipid | 1.33E-02 | Increased | 2,17 | 9 |
| Molecular Transport | secretion of molecule | 2.84E-03 | Increased | 4,248 | 24 |
| Molecular Transport | transport of molecule | 2.26E-03 | Increased | 3,621 | 58 |
| Molecular Transport | secretion of steroid | 8.81E-03 | Increased | 2,183 | 7 |
| Molecular Transport | transport of lipid | 1.33E-02 | Increased | 2,17 | 9 |
| Protein Degradation | degradation of protein | 2.70E-03 | Increased | 2,594 | 22 |
| Protein Synthesis | metabolism of protein | 8.20E-05 | Increased | 2,888 | 40 |
| Protein Synthesis | degradation of protein | 2.70E-03 | Increased | 2,594 | 22 |
| Small Molecule Biochemistry | fatty acid metabolism | 2.50E-03 | Increased | 2,567 | 25 |
| Small Molecule Biochemistry | secretion of steroid | 8.81E-03 | Increased | 2,183 | 7 |
| Small Molecule Biochemistry | transport of lipid | 1.33E-02 | Increased | 2,17 | 9 |
| Cell Morphology | contractility of muscle cells | 9.54E-03 | Decreased | -2 | 4 |

B) Functions – Plateau vs Atretic contrast

| **Category** | **Diseases or Functions Annotation** | **p-Value** | **Predicted Activation State** | **Activation z-score** | **# Molecules** |
| --- | --- | --- | --- | --- | --- |
| Cell Cycle | ploidy of cells | 1.17E-04 | Increased | 2,803 | 19 |
| Cell Cycle | ploidy | 1.05E-04 | Increased | 2,93 | 22 |
| Cell Death and Survival | cell death of epithelial cells | 3.55E-03 | Increased | 2,073 | 42 |
| Cell Death and Survival | cell death of lymphocytes | 1.45E-04 | Increased | 2,134 | 44 |
| Cell Death and Survival | cell death of carcinoma cell lines | 3.70E-03 | Increased | 2,145 | 22 |
| Cell Death and Survival | apoptosis of leukocytes | 3.02E-03 | Increased | 2,371 | 45 |
| Cell Death and Survival | apoptosis of colon cancer cell lines | 8.03E-04 | Increased | 2,703 | 26 |
| Dermatological Diseases and Conditions | Dermatitis | 6.44E-07 | Increased | 2,054 | 55 |
| Inflammatory Disease | Dermatitis | 6.44E-07 | Increased | 2,054 | 55 |
| Inflammatory Response | Dermatitis | 6.44E-07 | Increased | 2,054 | 55 |
| Lipid Metabolism | metabolism of membrane lipid derivative | 2.63E-03 | Increased | 2,143 | 39 |
| Organismal Survival | organismal death | 3.46E-05 | Increased | 2,29 | 215 |
| Small Molecule Biochemistry | metabolism of membrane lipid derivative | 2.63E-03 | Increased | 2,143 | 39 |
| Tissue Morphology | quantity of tumor cell lines | 7.89E-05 | Increased | 2,223 | 20 |
| Cancer | Cancer | 5.35E-08 | Decreased | -2,398 | 555 |
| Cancer | solid tumor | 2.31E-06 | Decreased | -2,179 | 473 |
| Cancer | growth of tumor | 1.29E-03 | Decreased | -2,091 | 42 |
| Cancer | development of tumor | 1.87E-03 | Decreased | -2,089 | 34 |
| Cardiovascular Disease | vascular disease | 2.96E-04 | Decreased | -2,082 | 77 |
| Cardiovascular System Development and Function | angiogenesis | 3.32E-03 | Decreased | -2,607 | 62 |
| Cardiovascular System Development and Function | development of vascular system | 3.02E-04 | Decreased | -2,4 | 97 |
| Cardiovascular System Development and Function | development of blood vessel | 5.56E-04 | Decreased | -2,234 | 76 |
| Cardiovascular System Development and Function | development of cardiovascular system | 4.25E-04 | Decreased | -2,201 | 96 |
| Cardiovascular System Development and Function | end diastolic pressure | 5.40E-04 | Decreased | -2,2 | 7 |
| Cardiovascular System Development and Function | end diastolic pressure of heart | 1.01E-03 | Decreased | -2,2 | 6 |
| Cell Cycle | entry into interphase of oocytes | 9.56E-06 | Decreased | -2 | 4 |
| Cell Death and Survival | cell survival | 7.89E-06 | Decreased | -3,457 | 133 |
| Cell Death and Survival | cell viability | 6.56E-06 | Decreased | -3,224 | 125 |
| Cell Death and Survival | cell viability of tumor cell lines | 3.84E-07 | Decreased | -2,682 | 83 |
| Cell Death and Survival | cell viability of myeloma cell lines | 3.83E-03 | Decreased | -2,143 | 11 |
| Cell-mediated Immune Response | transmigration of T lymphocytes | 3.28E-03 | Decreased | -2,176 | 7 |
| Cellular Assembly and Organization | organization of cytoplasm | 1.09E-10 | Decreased | -3,045 | 151 |
| Cellular Assembly and Organization | organization of cytoskeleton | 2.22E-11 | Decreased | -3,04 | 144 |
| Cellular Assembly and Organization | microtubule dynamics | 1.04E-09 | Decreased | -2,938 | 122 |
| Cellular Assembly and Organization | alignment of chromosomes | 9.69E-07 | Decreased | -2,792 | 10 |
| Cellular Assembly and Organization | formation of filaments | 2.82E-05 | Decreased | -2,649 | 46 |
| Cellular Assembly and Organization | formation of cytoskeleton | 7.88E-05 | Decreased | -2,457 | 41 |
| Cellular Assembly and Organization | development of cytoplasm | 2.71E-04 | Decreased | -2,457 | 46 |
| Cellular Development | proliferation of breast cancer cell lines | 2.22E-04 | Decreased | -4,099 | 43 |
| Cellular Development | proliferation of tumor cell lines | 2.24E-07 | Decreased | -2,962 | 149 |
| Cellular Development | proliferation of hepatoma cell lines | 1.02E-04 | Decreased | -2,738 | 23 |
| Cellular Development | proliferation of tumor cells | 3.17E-03 | Decreased | -2,253 | 46 |
| Cellular Function and Maintenance | organization of cytoplasm | 1.09E-10 | Decreased | -3,045 | 151 |
| Cellular Function and Maintenance | organization of cytoskeleton | 2.22E-11 | Decreased | -3,04 | 144 |
| Cellular Function and Maintenance | microtubule dynamics | 1.04E-09 | Decreased | -2,938 | 122 |
| Cellular Function and Maintenance | function of blood cells | 3.01E-04 | Decreased | -2,395 | 62 |
| Cellular Function and Maintenance | function of leukocytes | 4.38E-04 | Decreased | -2,395 | 57 |
| Cellular Growth and Proliferation | proliferation of breast cancer cell lines | 2.22E-04 | Decreased | -4,099 | 43 |
| Cellular Growth and Proliferation | proliferation of tumor cell lines | 2.24E-07 | Decreased | -2,962 | 149 |
| Cellular Growth and Proliferation | proliferation of cells | 2.85E-13 | Decreased | -2,749 | 343 |
| Cellular Growth and Proliferation | proliferation of hepatoma cell lines | 1.02E-04 | Decreased | -2,738 | 23 |
| Cellular Growth and Proliferation | proliferation of tumor cells | 3.17E-03 | Decreased | -2,253 | 46 |
| Cellular Movement | cell movement | 2.69E-07 | Decreased | -3,449 | 192 |
| Cellular Movement | migration of cells | 7.85E-07 | Decreased | -3,409 | 173 |
| Cellular Movement | cell movement of tumor cell lines | 4.82E-04 | Decreased | -3,326 | 71 |
| Cellular Movement | migration of tumor cell lines | 1.23E-03 | Decreased | -2,902 | 56 |
| Cellular Movement | invasion of cells | 3.99E-06 | Decreased | -2,858 | 81 |
| Cellular Movement | migration of breast cancer cell lines | 2.60E-03 | Decreased | -2,775 | 22 |
| Cellular Movement | cell movement of breast cancer cell lines | 1.55E-03 | Decreased | -2,617 | 26 |
| Cellular Movement | invasion of tumor cell lines | 8.79E-04 | Decreased | -2,5 | 53 |
| Cellular Movement | transmigration of T lymphocytes | 3.28E-03 | Decreased | -2,176 | 7 |
| DNA Replication, Recombination, and Repair | alignment of chromosomes | 9.69E-07 | Decreased | -2,792 | 10 |
| Hematological System Development and Function | end diastolic pressure | 5.40E-04 | Decreased | -2,2 | 7 |
| Hematological System Development and Function | end diastolic pressure of heart | 1.01E-03 | Decreased | -2,2 | 6 |
| Hematological System Development and Function | transmigration of T lymphocytes | 3.28E-03 | Decreased | -2,176 | 7 |
| Immune Cell Trafficking | transmigration of T lymphocytes | 3.28E-03 | Decreased | -2,176 | 7 |
| Organismal Development | development of blood vessel | 5.56E-04 | Decreased | -2,234 | 76 |
| Reproductive System Development and Function | entry into interphase of oocytes | 9.56E-06 | Decreased | -2 | 4 |
| Tissue Development | formation of filaments | 2.82E-05 | Decreased | -2,649 | 46 |
| Tumor Morphology | proliferation of tumor cells | 3.17E-03 | Decreased | -2,253 | 46 |
