# Supplementary material for: Global gene expression in granulosa cells of growing, plateau and atretic dominant follicles in cattle
Source: Reprod Biol Endocrinol. 2015 Mar 8;13:17. doi: 10.1186/s12958-015-0010-7 (PMC4355352; doi:10.1186/s12958-015-0010-7)
Supplement: Additional file 6: Table S4. — Upstream analysis in Ingenuity Pathway Analysis (IPA) software for the Growing vs. Plateau (A) and the Plateau vs Atretic (B) contrasts. [file 12958_2015_10_MOESM6_ESM.doc]

**Additional file 6.** **Upstream analysis in Ingenuity Pathway Analysis (IPA) software for the Growing vs. Plateau (A) and the Plateau vs Atretic (B) contrasts**. Only regulators with an Activation z-score greater than the absolute value of two are presented.

1. Upstream regulators – Growing vs Plateau contrast

| **Upstream Regulator** | **Fold Change** | **Molecule Type** | **Predicted Activation State** | **Activation z-score** | **p-value of overlap** |
| --- | --- | --- | --- | --- | --- |
| doxorubicin |  | chemical drug | Activated | 3,267 | 1.02E-05 |
| CD 437 |  | chemical drug | Activated | 3,051 | 3.67E-04 |
| CSF2 | 1.054 | cytokine | Activated | 2,913 | 4.13E-02 |
| phorbol myristate acetate |  | chemical drug | Activated | 2,884 | 8.23E-02 |
| SRF | 1.158 | transcription regulator | Activated | 2,747 | 4.96E-02 |
| STAT1 | 1.033 | transcription regulator | Activated | 2,607 | 1.43E-01 |
| NFkB (complex) |  | complex | Activated | 2,555 | 1.20E-01 |
| TP53 | -1.112 | transcription regulator | Activated | 2,486 | 2.34E-04 |
| topotecan |  | chemical drug | Activated | 2,449 | 2.15E-02 |
| TLR4 | 1.190 | transmembrane receptor | Activated | 2,423 | 1.73E-02 |
| Tgf beta |  | group | Activated | 2,395 | 4.15E-02 |
| IL6 | 1.052 | cytokine | Activated | 2,394 | 7.98E-03 |
| LDL |  | complex | Activated | 2,266 | 1.08E-03 |
| GnRH-A |  | chemical reagent | Activated | 2,19 | 1.71E-02 |
| TGFB3 | 1.279 | growth factor | Activated | 2,19 | 9.65E-03 |
| bleomycin |  | chemical drug | Activated | 2,156 | 1.25E-02 |
| TLR3 | -1.062 | transmembrane receptor | Activated | 2,155 | 9.09E-03 |
| calcitriol |  | chemical drug | Activated | 2,126 | 1.74E-02 |
| lipopolysaccharide |  | chemical drug | Activated | 2,117 | 2.06E-03 |
| TGFB1 | -1.035 | growth factor | Activated | 2,029 | 4.32E-04 |
| D-glucose |  | chemical - endogenous mammalian | Activated | 2,011 | 1.55E-02 |
| 17-alpha-ethinylestradiol |  | chemical drug | Activated | 2 | 1.82E-01 |
| IFNB1 | 1.010 | cytokine | Activated | 2 | 4.25E-01 |
| methylmercury |  | chemical toxicant | Inhibited | -2 | 4.65E-02 |
| ALDH1A2 | 1.017 | enzyme | Inhibited | -2 | 3.74E-03 |
| Immunoglobulin |  | complex | Inhibited | -2,076 | 8.16E-02 |
| Alpha catenin |  | group | Inhibited | -2,415 | 2.98E-02 |
| MYC | 2.431 | transcription regulator | Inhibited | -2,769 | 3.18E-04 |

1. Upstream regulators – Plateau vs Atretic contrast

| **Upstream Regulator** | **Fold Change** | **Molecule Type** | **Predicted Activation State** | **Activation z-score** | **p-value of overlap** |
| --- | --- | --- | --- | --- | --- |
| let-7 | 1.008 | microRNA | Activated | 4.615 | 3.87E-11 |
| U0126 |  | chemical - kinase inhibitor | Activated | 4.079 | 6.07E-07 |
| medroxyprogesterone acetate |  | chemical drug | Activated | 3.272 | 1.16E-10 |
| RBL2 | 1.207 | other | Activated | 3.264 | 1.70E-06 |
| LY294002 |  | chemical - kinase inhibitor | Activated | 3.229 | 9.07E-09 |
| calcitriol |  | chemical drug | Activated | 3.204 | 5.55E-08 |
| PD98059 |  | chemical - kinase inhibitor | Activated | 3.196 | 2.64E-05 |
| CD3 |  | complex | Activated | 3.168 | 1.28E-03 |
| triamcinolone acetonide |  | chemical drug | Activated | 3.165 | 3.60E-04 |
| SB203580 |  | chemical - kinase inhibitor | Activated | 3.156 | 2.87E-03 |
| CDKN2A | 1.036 | transcription regulator | Activated | 3.073 | 3.77E-06 |
| IRGM |  | other | Activated | 3.051 | 2.13E-07 |
| CD28 | -1.045 | transmembrane receptor | Activated | 3.018 | 8.65E-03 |
| MYCN | -1.013 | transcription regulator | Activated | 2.890 | 3.58E-22 |
| let-7a-5p (and other miRNAs w/seed GAGGUAG) |  | mature microRNA | Activated | 2.874 | 7.69E-02 |
| RBL1 |  | transcription regulator | Activated | 2.789 | 6.52E-06 |
| Alpha catenin |  | group | Activated | 2.749 | 3.66E-02 |
| PTEN | 1.273 | phosphatase | Activated | 2.730 | 3.29E-05 |
| NUPR1 | 1.008 | transcription regulator | Activated | 2.714 | 1.25E-05 |
| miR-1 (and other miRNAs w/seed GGAAUGU) |  | mature microRNA | Activated | 2.708 | 4.61E-03 |
| curcumin |  | chemical drug | Activated | 2.663 | 1.37E-02 |
| herbimycin |  | chemical - kinase inhibitor | Activated | 2.621 | 8.15E-03 |
| tyrphostin AG 1478 |  | chemical - kinase inhibitor | Activated | 2.613 | 5.33E-02 |
| miR-34a-5p (and other miRNAs w/seed GGCAGUG) |  | mature microRNA | Activated | 2.604 | 1.17E-02 |
| S-adenosylmethionine |  | chemical - endogenous mammalian | Activated | 2.567 | 8.28E-04 |
| Rb |  | group | Activated | 2.562 | 1.35E-05 |
| COL18A1 | -1.338 | other | Activated | 2.554 | 1.04E-03 |
| INSIG1 | -1.227 | other | Activated | 2.546 | 4.79E-02 |
| BNIP3L | 1.395 | other | Activated | 2.514 | 2.99E-06 |
| ABCB4 |  | transporter | Activated | 2.415 | 3.73E-03 |
| valsartan |  | chemical drug | Activated | 2.393 | 1.27E-02 |
| TCF3 |  | transcription regulator | Activated | 2.356 | 1.04E-04 |
| rottlerin |  | chemical toxicant | Activated | 2.346 | 9.35E-02 |
| BMS-690514 |  | chemical drug | Activated | 2.333 | 4.05E-05 |
| KDM5B | 1.302 | transcription regulator | Activated | 2.333 | 3.67E-05 |
| AG490 |  | chemical - kinase inhibitor | Activated | 2.307 | 4.25E-03 |
| PP2/AG1879 tyrosine kinase inhibitor |  | chemical - kinase inhibitor | Activated | 2.266 | 3.92E-04 |
| spironolactone |  | chemical drug | Activated | 2.236 | 1.96E-01 |
| 2,4,5,2',4',5'-hexachlorobiphenyl |  | chemical toxicant | Activated | 2.236 | 2.27E-01 |
| IgG |  | complex | Activated | 2.228 | 1.27E-03 |
| DACH1 | 1.396 | transcription regulator | Activated | 2.219 | 7.76E-03 |
| TP53 | 1.038 | transcription regulator | Activated | 2.215 | 2.12E-23 |
| triptolide |  | chemical drug | Activated | 2.186 | 1.26E-01 |
| wortmannin |  | chemical - kinase inhibitor | Activated | 2.183 | 2.32E-06 |
| baicalein |  | chemical - endogenous non-mammalian | Activated | 2.177 | 3.28E-02 |
| vitamin E |  | chemical drug | Activated | 2.158 | 2.04E-03 |
| SP600125 |  | chemical - kinase inhibitor | Activated | 2.125 | 2.22E-04 |
| epigallocatechin-gallate |  | chemical drug | Activated | 2.083 | 1.73E-03 |
| flavopiridol |  | chemical drug | Activated | 2.061 | 4.12E-03 |
| miR-16-5p (and other miRNAs w/seed AGCAGCA) |  | mature microRNA | Activated | 2.042 | 3.42E-02 |
| RB1 | 1.249 | transcription regulator | Activated | 2.036 | 1.72E-09 |
| troglitazone |  | chemical drug | Activated | 2.032 | 2.55E-06 |
| beta-carotene |  | chemical - endogenous mammalian | Activated | 2.000 | 1.33E-01 |
| bisindolylmaleimide |  | chemical - kinase inhibitor | Activated | 2.000 | 4.71E-02 |
| prazosin |  | chemical drug | Activated | 2.000 | 3.89E-03 |
| roscovitine |  | chemical drug | Activated | 2.000 | 4.08E-02 |
| fluocinolone acetonide |  | chemical drug | Activated | 2.000 | 1.63E-01 |
| ethidium |  | chemical toxicant | Activated | 2.000 | 1.14E-04 |
| rhodamine 6G |  | chemical toxicant | Activated | 2.000 | 4.73E-04 |
| miR-24-3p (and other miRNAs w/seed GGCUCAG) |  | mature microRNA | Activated | 2.000 | 1.64E-02 |
| IRF2 | 1.050 | transcription regulator | Activated | 2.000 | 9.32E-02 |
| CSF2 | -1.037 | cytokine | Inhibited | -6.465 | 7.53E-09 |
| TGFB1 | -1.088 | growth factor | Inhibited | -5.284 | 2.01E-12 |
| HGF | -1.019 | growth factor | Inhibited | -4.322 | 4.46E-13 |
| IL5 | -1.018 | cytokine | Inhibited | -4.300 | 4.78E-09 |
| PDGF BB |  | complex | Inhibited | -4.199 | 2.69E-12 |
| lipopolysaccharide |  | chemical drug | Inhibited | -4.185 | 7.20E-05 |
| IGF1 | -1.180 | growth factor | Inhibited | -4.130 | 1.32E-04 |
| Vegf |  | group | Inhibited | -3.952 | 1.23E-13 |
| ERBB2 |  | kinase | Inhibited | -3.785 | 1.51E-13 |
| mibolerone |  | chemical drug | Inhibited | -3.716 | 6.55E-06 |
| NFkB (complex) |  | complex | Inhibited | -3.698 | 4.80E-02 |
| Jnk |  | group | Inhibited | -3.641 | 3.67E-02 |
| EGF | -1.114 | growth factor | Inhibited | -3.630 | 3.07E-09 |
| FOXM1 | -1.014 | transcription regulator | Inhibited | -3.564 | 8.16E-09 |
| IL6 | -1.072 | cytokine | Inhibited | -3.426 | 5.14E-05 |
| TBX2 | 1.077 | transcription regulator | Inhibited | -3.281 | 3.42E-12 |
| ERK |  | group | Inhibited | -3.247 | 7.30E-06 |
| ANGPT2 | 4.629 | growth factor | Inhibited | -3.227 | 3.86E-02 |
| ionomycin |  | chemical reagent | Inhibited | -3.212 | 6.03E-02 |
| MTPN | -1.469 | transcription regulator | Inhibited | -3.138 | 1.17E-02 |
| SRF | -1.114 | transcription regulator | Inhibited | -3.019 | 2.79E-03 |
| FOXO1 | 1.187 | transcription regulator | Inhibited | -2.997 | 4.66E-05 |
| estrogen |  | chemical drug | Inhibited | -2.983 | 1.28E-06 |
| CD38 | -1.027 | enzyme | Inhibited | -2.965 | 2.13E-07 |
| SMAD4 | 1.087 | transcription regulator | Inhibited | -2.965 | 1.30E-02 |
| Notch |  | group | Inhibited | -2.954 | 3.36E-04 |
| F2 | 1.186 | peptidase | Inhibited | -2.938 | 9.24E-06 |
| anisomycin |  | chemical - endogenous non-mammalian | Inhibited | -2.924 | 6.73E-07 |
| PRL | 1.077 | cytokine | Inhibited | -2.905 | 8.39E-05 |
| Tgf beta |  | group | Inhibited | -2.894 | 1.60E-03 |
| isoproterenol |  | chemical drug | Inhibited | -2.883 | 1.60E-02 |
| kainic acid |  | chemical toxicant | Inhibited | -2.851 | 2.25E-03 |
| FGF1 | 1.049 | growth factor | Inhibited | -2.826 | 2.31E-03 |
| IL4 | -1.100 | cytokine | Inhibited | -2.825 | 9.17E-05 |
| POMC | 1.490 | other | Inhibited | -2.825 | 2.91E-03 |
| SREBF1 | 1.019 | transcription regulator | Inhibited | -2.782 | 4.55E-02 |
| ERK1/2 |  | group | Inhibited | -2.779 | 1.80E-04 |
| ITGAV | -1.015 | ion channel | Inhibited | -2.736 | 4.29E-07 |
| tetracycline |  | chemical drug | Inhibited | -2.730 | 4.58E-02 |
| SMAD3 | 1.058 | transcription regulator | Inhibited | -2.691 | 4.17E-04 |
| CREB1 | 1.070 | transcription regulator | Inhibited | -2.689 | 2.09E-05 |
| EDN1 | -1.103 | cytokine | Inhibited | -2.677 | 1.37E-02 |
| EGR1 | -2.743 | transcription regulator | Inhibited | -2.672 | 1.77E-02 |
| camptothecin |  | chemical toxicant | Inhibited | -2.657 | 3.24E-04 |
| Mek |  | group | Inhibited | -2.653 | 5.98E-03 |
| GnRH-A |  | chemical reagent | Inhibited | -2.607 | 9.82E-05 |
| 17-alpha-ethinylestradiol |  | chemical drug | Inhibited | -2.606 | 6.60E-04 |
| KITLG | 1.064 | growth factor | Inhibited | -2.601 | 7.53E-05 |
| TLR7 | -1.078 | transmembrane receptor | Inhibited | -2.596 | 2.59E-01 |
| E. coli B5 lipopolysaccharide |  | chemical - endogenous non-mammalian | Inhibited | -2.588 | 1.69E-01 |
| peptidoglycan |  | chemical - endogenous non-mammalian | Inhibited | -2.586 | 2.51E-01 |
| IL3 | -1.002 | cytokine | Inhibited | -2.585 | 5.23E-05 |
| KLF5 | -1.101 | transcription regulator | Inhibited | -2.573 | 1.17E-02 |
| Insulin |  | group | Inhibited | -2.563 | 1.35E-02 |
| AGN194204 |  | chemical drug | Inhibited | -2.560 | 1.42E-07 |
| 1,4-bis[2-(3,5-dichloropyridyloxy)]benzene |  | chemical toxicant | Inhibited | -2.554 | 2.97E-03 |
| NFKBIA | 1.073 | transcription regulator | Inhibited | -2.553 | 1.60E-02 |
| MYB | -1.922 | transcription regulator | Inhibited | -2.549 | 1.16E-03 |
| CCND1 | 1.049 | other | Inhibited | -2.540 | 1.61E-10 |
| nicotine |  | chemical drug | Inhibited | -2.470 | 2.92E-02 |
| AGT | -1.035 | growth factor | Inhibited | -2.469 | 1.48E-06 |
| FN1 | -1.522 | enzyme | Inhibited | -2.452 | 2.81E-03 |
| STAT3 | -1.156 | transcription regulator | Inhibited | -2.419 | 1.07E-01 |
| clozapine |  | chemical drug | Inhibited | -2.416 | 6.17E-02 |
| TNFSF11 | 1.025 | cytokine | Inhibited | -2.414 | 1.33E-02 |
| sphingosine-1-phosphate |  | chemical - endogenous mammalian | Inhibited | -2.412 | 6.52E-02 |
| carbamylcholine |  | chemical drug | Inhibited | -2.412 | 1.45E-02 |
| HTT |  | transcription regulator | Inhibited | -2.412 | 4.03E-03 |
| IL2 | -1.060 | cytokine | Inhibited | -2.407 | 1.29E-02 |
| PDGF-AA |  | complex | Inhibited | -2.400 | 4.59E-04 |
| PIM1 | -1.195 | kinase | Inhibited | -2.400 | 7.26E-03 |
| STAT5B | -1.016 | transcription regulator | Inhibited | -2.388 | 1.03E-02 |
| MYBL2 | -1.137 | other | Inhibited | -2.369 | 6.14E-04 |
| ELK1 | -1.222 | transcription regulator | Inhibited | -2.369 | 2.57E-03 |
| cyclophosphamide |  | chemical drug | Inhibited | -2.366 | 2.36E-01 |
| TSH |  | complex | Inhibited | -2.364 | 8.32E-03 |
| C5 |  | cytokine | Inhibited | -2.364 | 4.62E-01 |
| PI3K (complex) |  | complex | Inhibited | -2.335 | 2.05E-02 |
| trovafloxacin |  | chemical drug | Inhibited | -2.333 | 9.77E-03 |
| Ca2+ |  | chemical - endogenous mammalian | Inhibited | -2.330 | 8.49E-05 |
| Cg |  | complex | Inhibited | -2.306 | 1.04E-07 |
| carbon tetrachloride |  | chemical toxicant | Inhibited | -2.289 | 9.17E-03 |
| PRKCE | -1.140 | kinase | Inhibited | -2.264 | 2.19E-03 |
| STAT5A | -1.068 | transcription regulator | Inhibited | -2.242 | 1.51E-01 |
| Ap1 |  | complex | Inhibited | -2.236 | 1.32E-01 |
| NTRK1 | -2.027 | kinase | Inhibited | -2.236 | 4.59E-04 |
| SMOC2 | 1.411 | other | Inhibited | -2.236 | 8.18E-06 |
| PDGFB | -1.059 | growth factor | Inhibited | -2.234 | 5.24E-03 |
| lysophosphatidic acid |  | chemical - other | Inhibited | -2.229 | 2.99E-04 |
| nitroarginine |  | chemical reagent | Inhibited | -2.229 | 1.50E-02 |
| IL1 |  | group | Inhibited | -2.216 | 3.67E-01 |
| TCF7 | -1.792 | transcription regulator | Inhibited | -2.216 | 2.03E-03 |
| phorbol esters |  | chemical - other | Inhibited | -2.213 | 9.68E-02 |
| phenacetin |  | chemical drug | Inhibited | -2.213 | 7.11E-02 |
| reactive oxygen species |  | chemical toxicant | Inhibited | -2.213 | 4.81E-01 |
| MAPK3 | -1.142 | kinase | Inhibited | -2.213 | 9.32E-02 |
| SMAD1 | 1.160 | transcription regulator | Inhibited | -2.213 | 7.26E-03 |
| Mapk |  | group | Inhibited | -2.209 | 3.59E-02 |
| olanzapine |  | chemical drug | Inhibited | -2.205 | 9.69E-03 |
| TRAF2 | 1.058 | enzyme | Inhibited | -2.200 | 4.58E-02 |
| phorbol 12,13-dibutyrate |  | chemical - endogenous non-mammalian | Inhibited | -2.197 | 3.67E-02 |
| fluoxetine |  | chemical drug | Inhibited | -2.196 | 3.17E-01 |
| chloropromazine |  | chemical drug | Inhibited | -2.190 | 4.12E-03 |
| Gm-csf |  | group | Inhibited | -2.190 | 1.26E-01 |
| MAP2K4 | 1.328 | kinase | Inhibited | -2.190 | 2.17E-02 |
| MAP2K7 | 1.031 | kinase | Inhibited | -2.186 | 2.61E-02 |
| RET | -1.046 | kinase | Inhibited | -2.177 | 4.51E-03 |
| Smad2/3-Smad4 |  | complex | Inhibited | -2.176 | 1.14E-03 |
| entinostat |  | chemical drug | Inhibited | -2.173 | 4.88E-02 |
| methylnitronitrosoguanidine |  | chemical toxicant | Inhibited | -2.173 | 1.74E-02 |
| EGFR | 1.115 | kinase | Inhibited | -2.170 | 1.75E-05 |
| IL18 | 1.121 | cytokine | Inhibited | -2.169 | 1.00E00 |
| PTK2 | -1.447 | kinase | Inhibited | -2.166 | 7.31E-04 |
| FGF2 | -1.107 | growth factor | Inhibited | -2.156 | 6.35E-06 |
| TLR3 | 1.616 | transmembrane receptor | Inhibited | -2.145 | 3.43E-01 |
| Growth hormone |  | group | Inhibited | -2.140 | 2.40E-03 |
| CCL5 | 1.141 | cytokine | Inhibited | -2.137 | 3.22E-02 |
| Pkc(s) |  | group | Inhibited | -2.131 | 2.27E-02 |
| GNRH |  | group | Inhibited | -2.123 | 2.65E-03 |
| 5-O-mycolyl-beta-araf-(1->2)-5-O-mycolyl-alpha-araf-(1->1')-glycerol |  | chemical - endogenous non-mammalian | Inhibited | -2.121 | 8.30E-02 |
| NEDD9 | -1.183 | other | Inhibited | -2.121 | 4.11E-04 |
| MAPK1 | 1.009 | kinase | Inhibited | -2.120 | 1.09E-02 |
| NFYA | -1.062 | transcription regulator | Inhibited | -2.111 | 1.01E-06 |
| IFNG | -1.030 | cytokine | Inhibited | -2.109 | 3.22E-04 |
| MAP3K1 | 1.205 | kinase | Inhibited | -2.106 | 2.04E-03 |
| E2F1 | -1.471 | transcription regulator | Inhibited | -2.100 | 1.27E-10 |
| gentamicin |  | chemical drug | Inhibited | -2.093 | 1.09E-02 |
| TLR9 | 1.034 | transmembrane receptor | Inhibited | -2.066 | 1.42E-01 |
| IL13 | -1.092 | cytokine | Inhibited | -2.065 | 5.13E-05 |
| Il3 |  | cytokine | Inhibited | -2.060 | 2.99E-02 |
| TNF | 1.019 | cytokine | Inhibited | -2.056 | 1.68E-04 |
| VEGFA | -1.235 | growth factor | Inhibited | -2.056 | 2.42E-04 |
| mono-(2-ethylhexyl)phthalate |  | chemical toxicant | Inhibited | -2.038 | 6.28E-03 |
| norepinephrine |  | chemical - endogenous mammalian | Inhibited | -2.036 | 1.01E-01 |
| okadaic acid |  | chemical toxicant | Inhibited | -2.024 | 8.30E-02 |
| HIF1A | 1.711 | transcription regulator | Inhibited | -2.023 | 6.71E-05 |
| PRKCD | -1.290 | kinase | Inhibited | -2.020 | 1.99E-02 |
| FOXL2 | 1.087 | transcription regulator | Inhibited | -2.020 | 2.85E-02 |
| valproic acid |  | chemical drug | Inhibited | -2.019 | 6.72E-05 |
| methotrexate |  | chemical drug | Inhibited | -2.010 | 4.06E-02 |
| T3-TR-RXR |  | complex | Inhibited | -2.000 | 1.73E-01 |
| RBX1 | 1.527 | enzyme | Inhibited | -2.000 | 1.10E-04 |
| XIAP | 1.029 | enzyme | Inhibited | -2.000 | 1.96E-02 |
| PGF | 1.348 | growth factor | Inhibited | -2.000 | 2.72E-02 |
| RARA | 1.260 | ligand-dependent nuclear receptor | Inhibited | -2.000 | 4.53E-01 |
| KAT5 | 1.476 | transcription regulator | Inhibited | -2.000 | 4.87E-01 |
| IGF2BP1 | 1.039 | translation regulator | Inhibited | -2.000 | 1.64E-02 |
